# Domain‐specific cognitive impairment in multiple sclerosis: A systematic review and meta‐analysis

**DOI:** 10.1002/acn3.51976

**Published:** 2024-01-11

**Authors:** Katalin Lugosi, Marie A Engh, Zsolt Huszár, Péter Hegyi, Péter Mátrai, Gábor Csukly, Zsolt Molnár, Klaudia Horváth, Dóra Mátis, Zsolt Mezei

**Affiliations:** ^1^ Centre for Translational Medicine Semmelweis University Üllői út 26 1085 Budapest Hungary; ^2^ Multiple Sclerosis Centre Bajcsy‐Zsilinszky Hospital Maglódi út 89‐91 Budapest 1106 Hungary; ^3^ Department of Psychiatry and Psychotherapy Semmelweis University Balassa utca 6 Budapest 1083 Hungary; ^4^ Institute of Pancreatic Diseases, Semmelweis University Budapest Hungary; ^5^ Institute for Translational Medicine, Medical School University of Pécs Pécs Hungary; ^6^ Department of Anaesthesiology and Intensive Therapy Semmelweis University Üllői út 78 1083 Budapest Hungary; ^7^ Department of Anaesthesiology and Intensive Therapy Poznan University of Medical Sciences ul. Przybyszewskiego 49 60‐355 Poznan Poland; ^8^ Department of Neurology Semmelweis University Balassa utca 6 1083 Budapest Hungary

## Abstract

**Objective:**

Methods of cognitive measurements in multiple sclerosis (MS) are not standardized. We aimed to identify the prevalence of cognitive domain‐specific impairment (DSI) in MS by using subtests of the Brief Repeatable Battery of Neuropsychological Tests (BRB‐N) with analyzing different cutoff values.

**Methods:**

The systematic review and meta‐analysis were registered on PROSPERO (ID: CRD42021287004). The systematic literature search was performed via PubMed, Embase, and CENTRAL on 24 October 2021. Inclusion criteria were adults of different MS subtypes (CIS, RRMS, PPMS, and SPMS) with the condition of distinct DSI measured by BRB‐N. Pediatric MS, computerized versions of BRB‐N, and patients receiving steroids were excluded. Primary outcome was pooled prevalence rates of impaired patients within each cutoff and MS subtype, with 95% confidence interval, I‐squared statistics for heterogeneity, and chi‐squared test for subgroup differences. Risk of bias was assessed using the “JBI Quality Assessment Tool for Prevalence Studies.”

**Results:**

In 48 eligible observational studies (*n* = 3431 patients), the three most prevalent thresholds were the 2.0 SD and 1.5 SD below the mean of normative values, and the score below the fifth percentile of the normative values. A progressively increasing worsening of the overall DSI was observed from CIS, moving toward RRMS, PPMS, and SPMS.

**Interpretation:**

Cognitive impairment is observed in all MS phenotypes, with varying degrees. Due to several potential influencing factors, our comprehensive literature review has not revealed consistent findings, and we, therefore, recommend considering a more sophisticated, “*individual referencing*” approach, acknowledging the diverse clinical and sociodemographic characteristics among populations and disparities in cognitive testing.

## Introduction

Cognitive impairment (CI) is one of the most common, life‐altering consequences of multiple sclerosis (MS), and it can occur independently of physical disability. The prevalence of CI in MS is estimated to range from 43% to 70%.^1^

The fifth revision of the Diagnostic and Statistical Manual of Mental Disorders (DSM‐5; published by the American Psychiatric Association)^2^ assesses the symptomatology of cognitive disorders along the six main cognitive domains (complex attention/information processing speed, memory/learning functions, language abilities, executive functions, perceptual‐motor/visuospatial abilities, and social cognition) by examining CI of various etiologies and grading them into “major” and “mild” severity. CI in MS is discussed in a subsection of “mild neurocognitive disorders.”

However, in “mild neurocognitive disorders,” a neuropsychological test (NPT) performance should be 1–2 SD below the normative values or between the 3rd and 16th percentile for tests, where appropriate norms are available,^3^ neither the currently available international literature nor the DSM‐5 manual provides recommended, standardized, internationally accepted neuropsychological tests and cutoff values for measuring cognitive domain‐specific impairment (DSI), which would presumably be essential for the exact, consistent definition of CI in MS.^4^

Nevertheless, beyond the scope of DSM‐V, there is a particular perception of CI in MS. Due to the various main clinical manifestations of the disease (relapsing–remitting or progressive) and the additional subclassifications of progressive forms (“active” or “not active”, “with,” or “without progression”),^5^ the assessment of cognitive function in MS requires a “continuous reflection strategy” that resonates with the changing clinical characteristics of the disease. This means that reassessments are needed to detect the influence of disease activity, clinical/radiological progression, relapses/recovery of relapses, treatment response, and patients' self‐reported cognitive complaints.^6^, ^7^

Although some evidence suggests that as “hallmark”^6^ or “core deficits,” IPS/attention and working memory are probably the most affected CDs early in the course of the disease^1^, ^8^ and accordingly, “as a minimum,” the Symbol Digit Modalities Test (SDMT) measuring IPS should be performed at baseline and every 2–3 years,^6^, ^7^ the exploration of domain‐specific impairments with a series of neuropsychological tests aligns best with the abovementioned reflective assessment strategy.

Three batteries of validated NPTs are widely accepted for the assessment of DSI: the Rao's Brief Repeatable Battery of Neuropsychological Tests (BRB‐N),^9^ the Minimal Assessment of Cognitive Functioning in Multiple Sclerosis (MACFIMS),^10^ and the Brief International Cognitive Assessment for MS (BICAMS).^11^

BRB‐N is one of the most sensitive, specific (71% sensitivity and 94% specificity),^9^ and widely used validated testing tools detecting five measurable parts of six main cognitive domains (CD). The Paced Auditory Serial Addition Test 3 (PASAT3)^12^ measures working memory; the Symbol Digit Modalities Test (SDMT)^13^ measures information processing speed (IPS) and complex attention; the Word List Generation Test (WLG)^14^ measures language function and verbal fluency; the 10/36 Spatial Recall Test (SPART)^15^ measures perceptual‐motor/visuospatial memory and refers to some aspects of executive functions; and the Selective Reminding Test (SRT)^16^ measures learning and verbal memory.

However, it is essential to recognize that an ongoing and perpetual debate surrounds the precise domain(s) assessed by these specific tests, moreover, the individual CDs overlap and mutually influence each other.^17^

For all these reasons, only a few studies have conducted comprehensive population‐level or meta‐analysis studies of cognitive domain‐specific impairment using sophisticated neuropsychological batteries. The results are also heterogeneous, despite the fact that this approach would provide the clearest picture of this aspect of MS.^18^

Planche et al., in their population‐based study, found that regardless of disease course, the IPS was the most frequently impaired cognitive domain, followed by verbal episodic memory, executive functions, visuospatial construction, verbal fluency, working memory, and language. However, they did not use a predefined and elaborate test battery, but a customized one. Patients with SPMS were more frequently affected than patients with LRRMS (late relapsing–remitting MS with a disease duration of more than 10 years) in all CDs except language.^18^

Potagas et al. provide a comprehensive picture of the cognitive DSI (exploring possible “pattern of cognitive impairment”) observed in different clinical subtypes of MS (RRMS, PPMS, and SPMS), including CIS patients. A further advantage is that it presents this through evaluating a standardized neuropsychological battery (BRB‐N). They found that except for the relatively spared (not significantly different from healthy controls) cognitive domain of verbal learning/memory (as measured by SRT) in CIS patients, a progressively worse cognitive impairment was observed for all cognitive domains along the CIS‐RRMS‐PPMS‐SPMS axis. Still, the study failed to detect different patterns of impairment between MS subtypes.^19^

Johnen et al. conducted a comparative analysis to assess the extent and characteristics of cognitive impairment (CI) as determined by standardized neuropsychological tests in patients with primary progressive multiple sclerosis (PPMS) as compared to relapsing–remitting multiple sclerosis (RRMS). A comprehensive non‐predefined neuropsychological battery was employed to evaluate 12 domains of dysfunction, including cognitive, manual dexterity, anxiety and depression, and fatigue. The study revealed that individuals with PPMS exhibited significantly more pronounced CI across all assessed CDs compared to those with RRMS. Notably, distinctions in verbal learning and memory were particularly prominent, establishing a clear demarcation between PPMS and RRMS irrespective of demographic variances.^20^

Accordingly, in order to clarify and organize the issues discussed above, our aim was to determine the prevalence of DSI in subtypes of MS, based on specific standardized NPTs, taking into account different cutoff values. We chose the subtests of BRB‐N battery, because it had been widely validated in several countries and it had relatively high sensitivity and specificity. Using this approach, our study is expected to contribute to unveiling research gaps or contradictions within the existing international literature, which could inspire further investigations and the development of international guidelines. Moreover, expanding our understanding in this field can facilitate interdisciplinary collaboration among related scientific areas, including neurology, clinical neuropsychology, psychiatry, and rehabilitation.

## Methods

### Study registration

Our analysis protocol was registered in PROSPERO (international database of prospectively registered systematic reviews; registration ID: CRD42021287004) which we followed without any deviations during the process. We followed the recommendations of the Cochrane Handbook^21^ and the Preferred Reporting Items for Systematic Reviews and Meta‐Analyses (PRISMA) 2020 statement^22^ (Supplementary Material, Appendix 1).

### Search strategy

The systematic search was performed within three major databases (Medline—via PubMed, Embase, and CENTRAL—The Cochrane Central Register of Controlled Trials) without restrictions on 24 October 2021 (Supplementary Material, Appendix 2). We searched the databases from the inception.

### Selection process

The selection was performed using Endnote 20 (Clarivate Analytics, Philadelphia, PA, USA) software. After automatic and manual removal of duplicates, a selection process was conducted by two independent review authors (KL and ZH) in two steps (by title and abstract, then by full‐text), with any disagreements resolved by a third author (ZMe). The degree of agreement was quantified by Cohen's kappa statistics.

The “CoCoPop” framework (i.e., condition – context – population)^23^ was used to define our selection criteria: the population included adult patients of both sexes (age ≥ 18 years) diagnosed with MS in the context of MS subtypes (i.e., CIS: clinically isolated syndrome, RRMS: relapsing–remitting MS, PPMS: primary progressive MS and SPMS: secondary progressive MS) according to the Lublin classifications^5^ with the condition of distinct DSI measured by subtests of the BRB‐N battery (Supplementary Material, Appendix 3).

We excluded studies that examined pediatric or pediatric‐onset MS (POMS) populations, computerized versions of BRB‐N subtests, and tested patients during relapse/steroid administration, as it could significantly alter cognitive test results.^24^

Diagnosis of MS was based on the McDonald criteria,^25^ and as this was first established in 2001, studies published before 2001 were eventually excluded (Supplementary Material, Appendix 4).

As no language restrictions were set, two eligible articles in languages other than English were included and translated using a translation tool (DeepL Translator),^26^ Only observational studies were eligible for analysis. The primary outcome was the prevalence (%) of DSI in clinical MS subtypes based on BRB‐N subtests.

As different cutoff values had been mentioned in the literature to define abnormal results in BRB‐N subtests, each cutoff value was analyzed individually.

### Data collection

Data extraction was performed by two reviewers independently (KL and ZH) and compared by a third author (ZMe). Baseline data, outcomes, and their definitions for studies and populations were extracted into a predesigned Excel (Microsoft Corporation, Redmond, Washington, USA) spreadsheet.

SRT also provides the short‐term and long‐term components of memory by the consistency of retrieval from long‐term memory (SRT LTS: SRT – long‐term storage and SRT CLTR: SRT – consistent long‐term retrieval) and delayed recall (SRT DR: SRT – delayed recall), which is the total number of words recalled after the delayed period.

The 10/36 SPART test has immediate (SPART) and delayed recall (SPART DR) subtests.

Where a study provided information on these subtests, these were also collected and analyzed.

### Statistical analysis

The statistical analysis of the data was conducted using the R programming language.^27^ We used the *meta* ^28^ package for calculations and plots.

For each MS subtype, test, and cutoff value, we extracted the number of MS patients and the number of those who were found to be impaired in the given CD. To conduct the statistical analysis, we chose the three most commonly used cutoff values: a cutoff of ≤1.5 standard deviations (SD) and ≤2.0 SD below the normative value and ≤ 5th percentile of the normative population (i.e., compared to the healthy control group). Patients with MS whose scores fell below these cutoff values were declared as impaired in the given CD. Raw prevalences from the selected studies were transformed to logit scale, then pooled using a random‐effects model and then transformed back to the original scale for data presentation.^29^ Ƭ^2^ was estimated using the restricted maximum likelihood method.^30^ Statistical heterogeneity across trials was assessed using the Cochrane Q test, and the I^2^ values.^31^ Forest plots were used to summarize results graphically. Where applicable, we reported the 95% prediction interval (i.e., the estimated range that contains 95% of true prevalence) following the recommendations.^32^

### Risk of bias assessment

Risk of bias was assessed using the Joanna Briggs Institute (JBI) Quality Assessment Tool for Prevalence Studies^33^ framework by two independent reviewers (KL and DM) with disagreements resolved by a third reviewer (ZMe).

## Results

### Selection and study characteristics

Our search key initially identified 14,031 articles, and eventually 48 studies were included in the synthesis (both in the systematic review and in the meta‐analysis).

**Figure 1:**
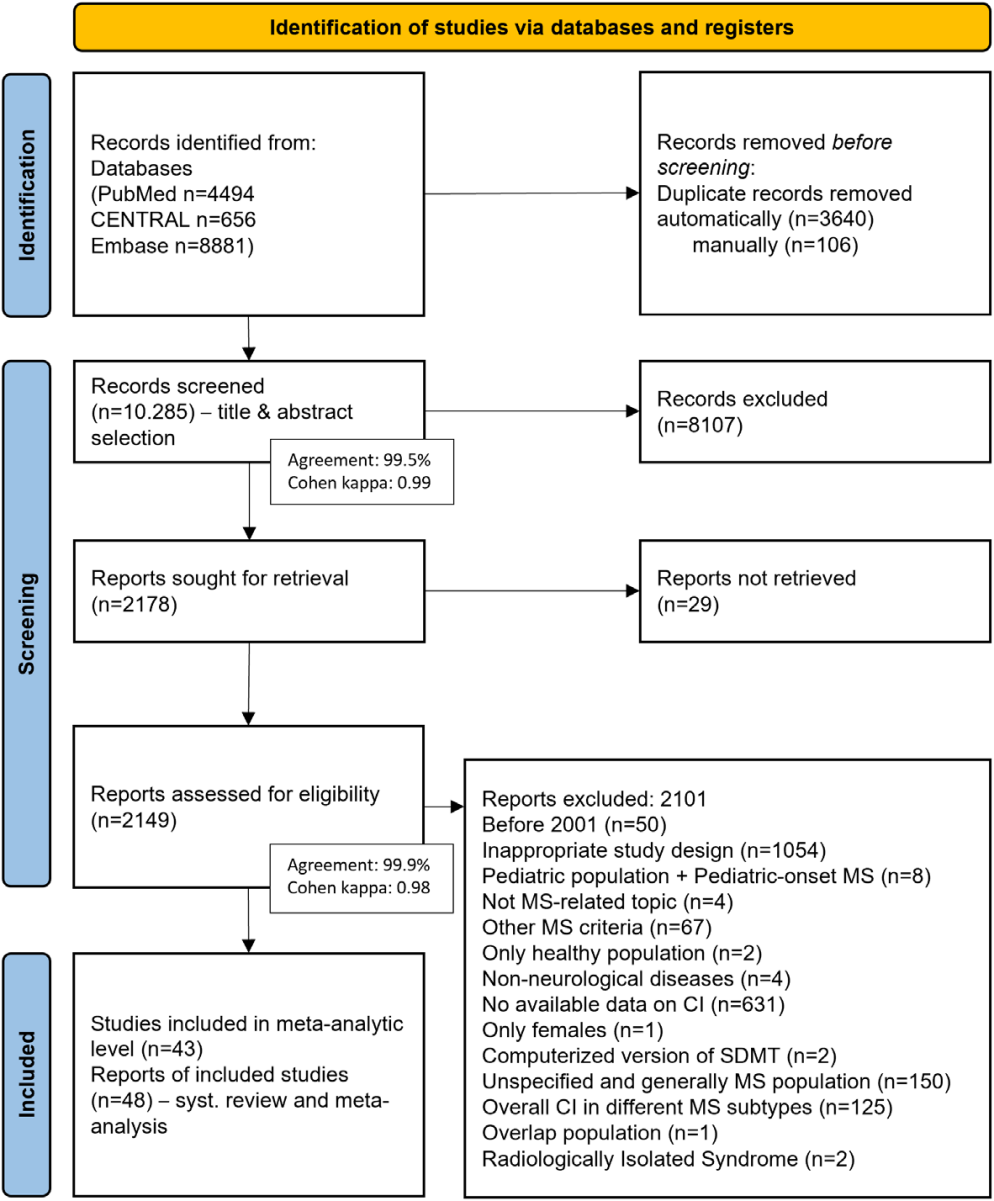
Flow diagram of study identification and selection by PRISMA 2020 with details of the reasons for exclusion (See also Supplementary Material, Appendix 4).

Details of the complete selection process are shown in the PRISMA flowchart (Fig. 1).

The abovementioned three most commonly used NPT cutoff values were included in our meta‐analysis, as they were suitable for quantitative analysis, and five additional studies were also reviewed systematically that did not qualify for our meta‐analysis as they did not adhere to our predefined cutoff values. No distinction was made between cutoff values in the score or calculated z score if they contained the same SD value, or between the prespecified “normative” or self‐reported “healthy controls” reference populations.

All included articles were observational studies. Where a longitudinal study was considered eligible, baseline results were used as cross‐sectional data.

Finally, the results of 3131 MS patients (450 CIS, 2393 RRMS, 134 PPMS and 154 SPMS) were included in the meta‐analysis, and a further 300 were included in the qualitative assessment only (18 CIS, 197 RRMS, 12 PPMS and 73 SPMS).

A list of the references for all included studies are available in the Supplementary Material, Appendix 5. Baseline characteristics of the included studies are detailed (Supplementary Material, Appendix 6, eTable 1).

### Evaluation of individual DSI across different MS subtypes based on separate cutoffs used to define impairment

#### A cutoff of 2.0 SD below the normative value

Due to a lack of data, at this cutoff value we were only able to perform a detailed analysis for all subtypes at PASAT‐3, while at all other tests, we only had data for CIS and RRMS.

**Figure 2:**
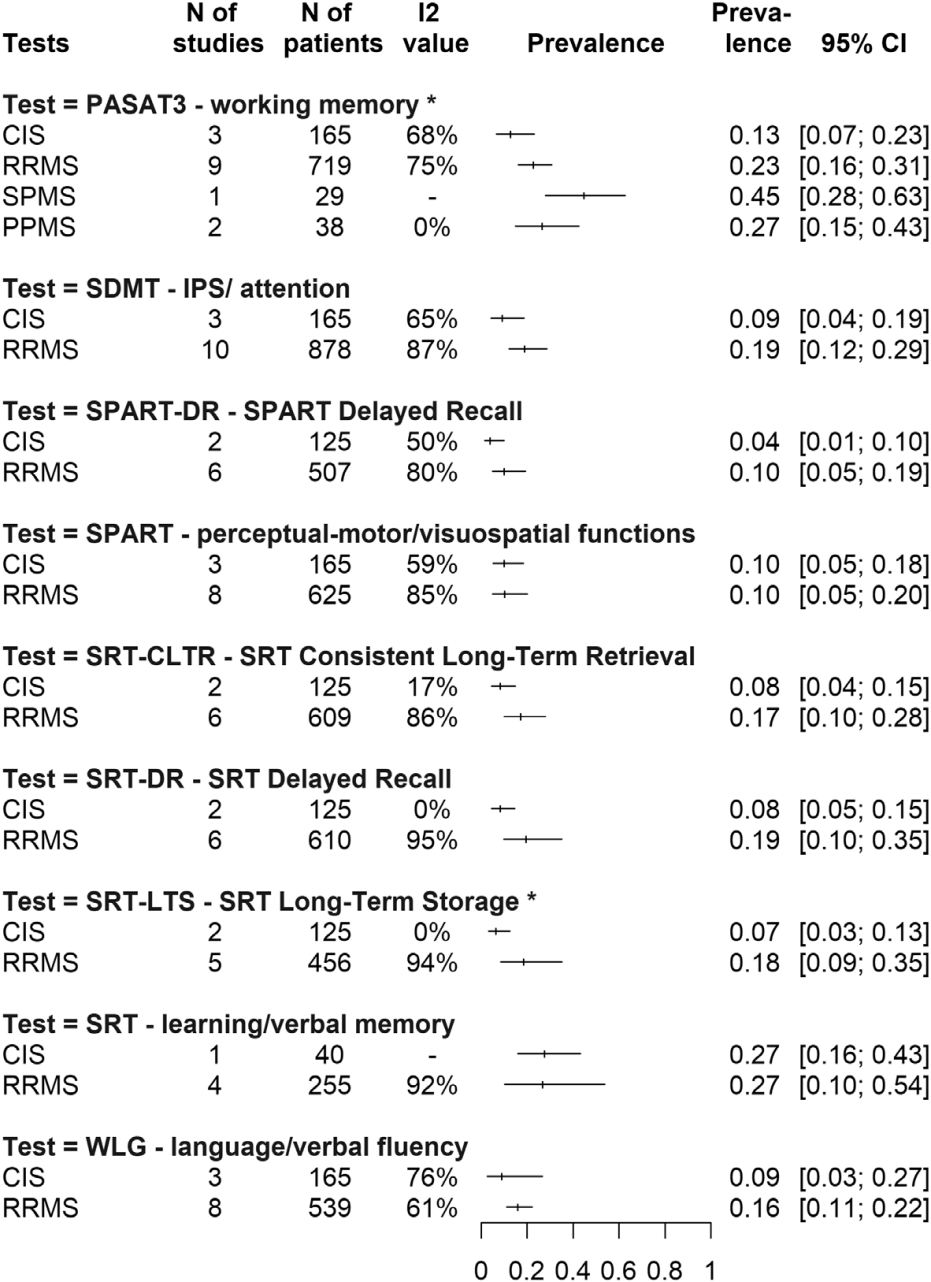
Summary graph of DSI (domain‐specific impairment) prevalence rates across the subtests of the BRB‐N (Brief Repeatable Battery of Neuropsychological Tests) battery at the “2.0 SD below the normative values” cutoff. On the left side of the figure are the subtests of the BRB‐N battery. Below each test, MS subtypes are shown. At this cutoff value, only the PASAT3 (Paced Auditory Serial Addition Test 3) subtest had sufficient data in the literature for all MS subtypes; for the other BRB‐N subtests only the CIS and RRMS subtypes were available. On the right side of the figure are the prevalence rates (with confidence intervals) of impaired patients grouped by MS subtype, for the given BRB‐N subtest, using the “2.0 SD below the normative values” cutoff.

Impaired working memory (PASAT3) affects **13%** CI: [0.07; 0.23] of CIS, **23%** CI: [0.16; 0.31] of RRMS, **27%** CI: [0.15; 0.43] of PPMS and **45%** CI: [0.28; 0.63] of SPMS patients, whereas impairment of delayed recall in visuospatial abilities (SPART DR) is present in only in **4%** CI: [0.01; 0.10] of CIS patients and **10%** CI: [0.05; 0.19] of RRMS patients. Impairment of IPS (SDMT) and decline in verbal fluency (WLG) also affect **9%** CI: [0.04; 0.19] **– 9%** CI: [0.03; 0.27] of CIS patients and are present in **19%** CI: [0.12; 0.29] and **16%** CI: [0.11; 0.22] of RRMS patients, respectively. Impairment of learning and verbal memory domains affects **7%–8%** (CI: [0.05; 0.15] at SRT DR, CI: [0.03; 0.13] at SRT LTS, CI: [0.04; 0.15] at SRT CLTR) of CIS patients and **17%–19%** (CI: [0.10; 0.35] at SRT DR, CI: [0.09; 0.35] at SRT LTS, CI: [0.10; 0.28] at SRT CLTR) of RRMS patients, depending on the SRT test recall. All these prevalence values can be found visually in the summary of Fig 2.

#### A cutoff of 1.5 SD below the normative value

At this cutoff value, working memory impairment (PASAT3) is present in only **4%** CI: [0.01; 0.11] of CIS patients, **20%** CI: [0.09; 0.38] of RRMS patients, **24%** CI: [0.07; 0.59] of PPMS and **31%** CI: [0.08; 0.70] of SPMS groups. IPS impairment (SDMT) affects **13%** CI: [0.08; 0.20] of CIS patients and **25%** CI: [0.18; 0.33] of RRMS patients, with PPMS and SPMS being affected by **35%** CI: [0.14; 0.63] and **61%** CI: [0.31; 0.85], respectively. Impairment of verbal fluency (WLG) affects almost the same proportion of PPMS and SPMS patients (**80%** CI: [0.64; 0.90] and **81%** CI: [0.63; 0.91], respectively), compared to **15%** CI: [0.02; 0.65] in CIS and **35%** CI: [0.25; 0.48] in RRMS. For visuospatial skills, there are sufficient data only for the impairment of delayed recall (SPART DR): almost equal in CIS and RRMS (**16%** CI: [0.09; 0.26] and **15%** CI: [0.08; 0.26] respectively), **57%** CI: [0.41; 0.72] in PPMS, and a very high proportion of patients in SPMS (**74%** CI: [0.56; 0.87]).

**Figure 3:**
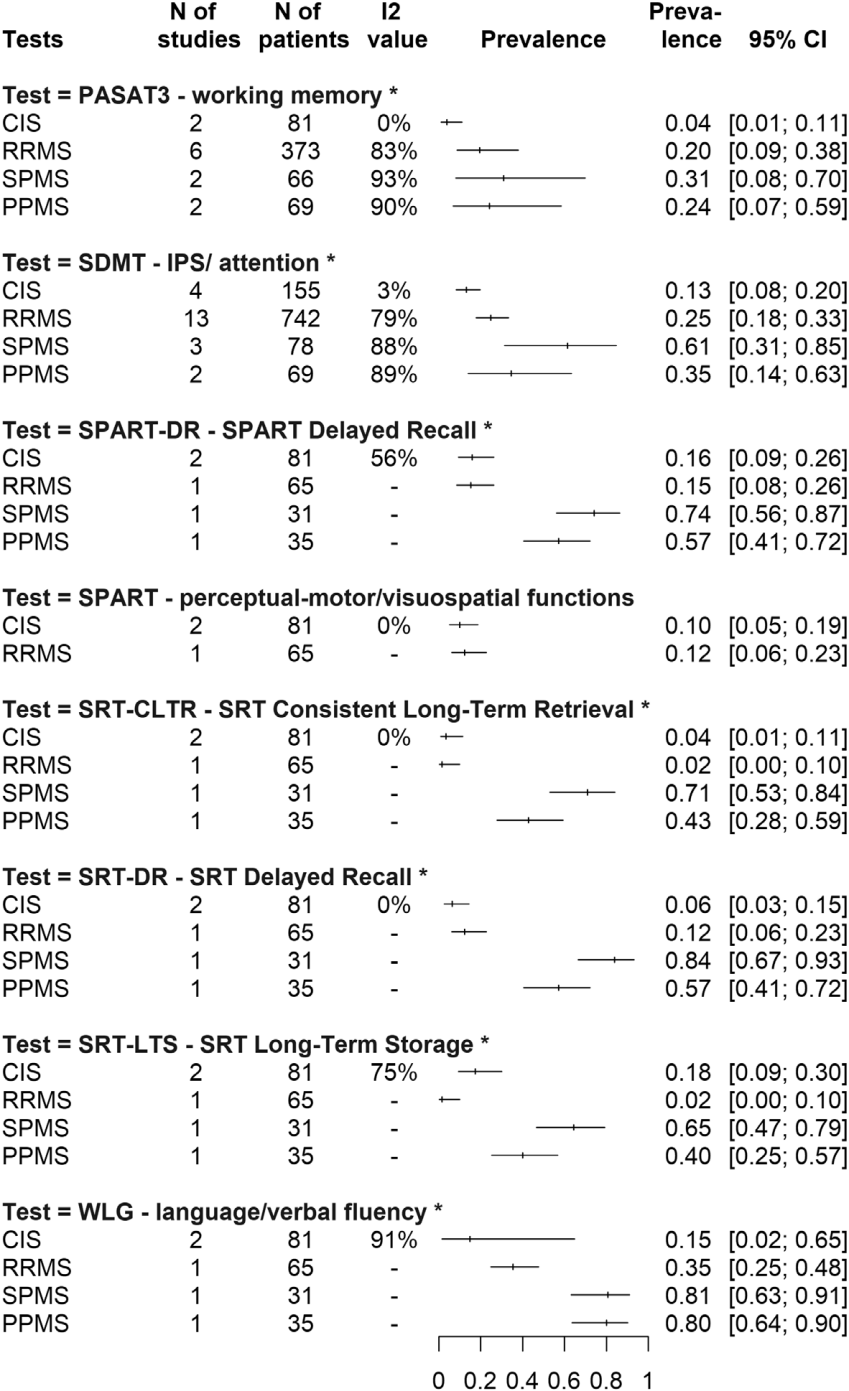
Summary graph of DSI (domain‐specific impairment) prevalence rates across the subtests of the BRB‐N (Brief Repeatable Battery of Neuropsychological Tests) battery at the “1.5 SD below the normative values” cutoff. On the left side of the figure are the subtests of the BRB‐N battery. Below each test, MS subtypes are shown. On the right side of the figure are the prevalence rates (with confidence intervals) of impaired patients grouped by MS subtype, for the given BRB‐N subtest, using the “1.5 SD below the normative values” cutoff.

Impairment of delayed recall of learning/verbal memory (SRT) is present only in **6%** CI: [0.03; 0.15] of CIS patients, **12%** CI: [0.06; 0.23] of RRMS patients, **57%** CI: [0.41; 0.72] of PPMS and **84%** CI: [0.67; 0.93] of SPMS group. All these prevalence values can be found visually in the summary of Figure 3.

#### A cutoff of the score below the fifth percentile of the normative values

At this cutoff value, IPS impairment (SDMT) affects **74%** CI: [0.60; 0.85] of SPMS patients, it is present in **59%** CI: [0.40; 0.76] of PPMS, it is the lowest in CIS (**19%** CI: [0.13; 0.26]) and the RRMS subtype is intermediate between the early form (CIS) and progressive forms, affecting **25%** (CI: [0.19; 0.32]) of patients.

**Figure 4:**
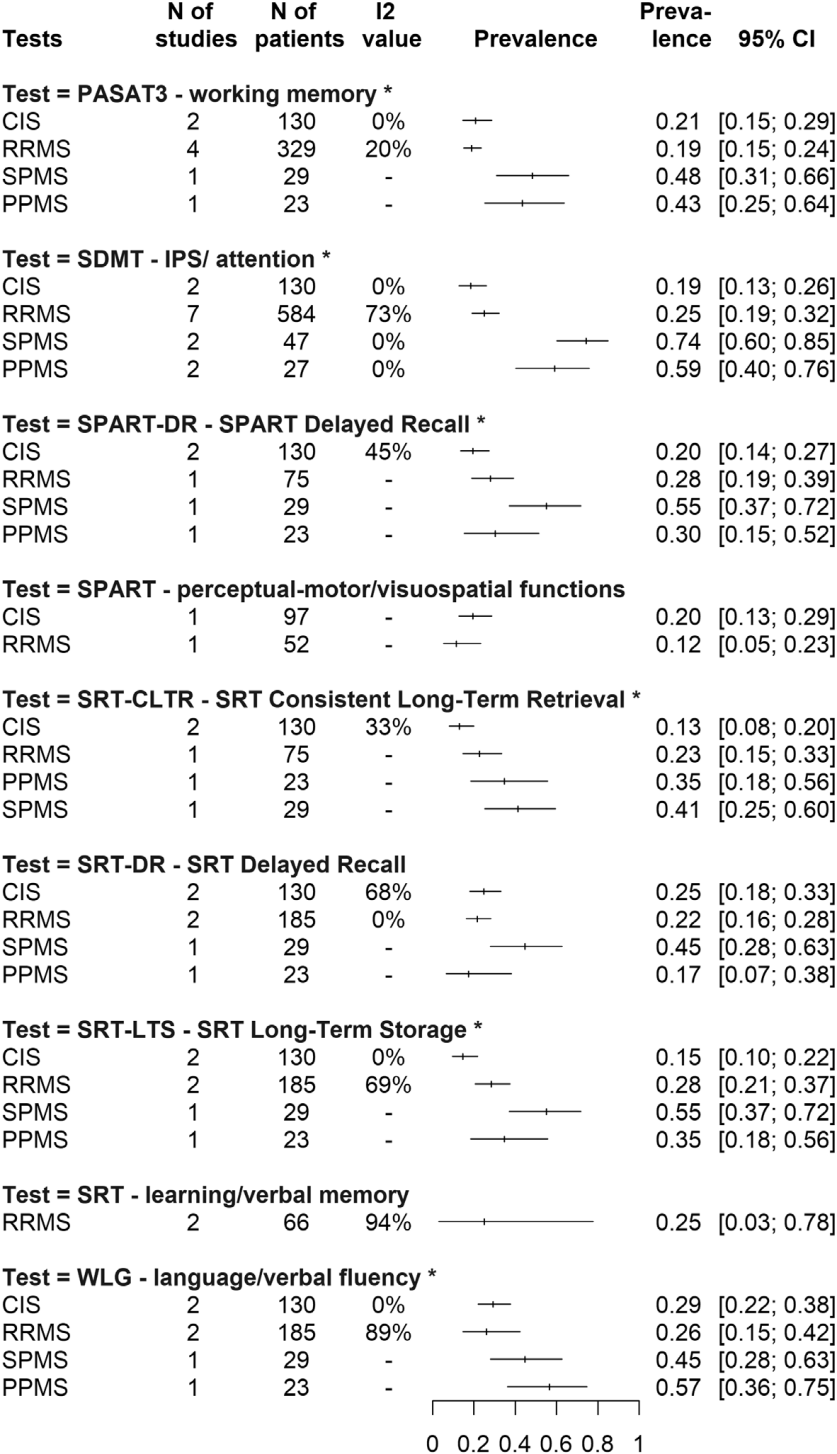
Summary graph of DSI (domain‐specific impairment) prevalence rates across the subtests of the BRB‐N (Brief Repeatable Battery of Neuropsychological Tests) battery at the “score below the fifth percentile of the normative values” cutoff. On the left side of the figure are the subtests of the BRB‐N battery. Below each test, MS subtypes are shown. On the right side of the figure are the prevalence rates (with confidence intervals) of impaired patients grouped by MS subtype, for the given BRB‐N subtest, using the “score below the fifth percentile of the normative values” cutoff.

In terms of working memory impairment (PASAT3), the CIS group shows a slightly higher rate compared to the RRMS group (**21%** CI: [0.15; 0.29] vs. **19%** CI: [0.15; 0.24]), followed by PPMS (**43%** CI: [0.25; 0.64]) and then the SPMS (**48%** CI: [0.31; 0.66]) subtype. For visuospatial memory impairment, only the delayed recall test (SPART DR) has sufficient information for all subtypes, with **20%** (CI: [0.14; 0.27]) of CIS patients, **28%** (CI: [0.19; 0.39]) of RRMS, **30%** (CI: [0.15; 0.52]) of PPMS and slightly more than half (**55%** CI: [0.37; 0.72]) of SPMS patients impaired. In terms of learning and verbal memory impairment, depending on the type of memory retrieval, **13%–25%** (CI: [0.18; 0.33] at SRT DR, CI: [0.10; 0.22] at SRT LTS, CI: [0.08; 0.20] at SRT CLTR) of CIS patients, **22%–28%** (CI: [0.16; 0.28] at SRT DR, CI: [0.21; 0.37] at SRT LTS, CI: [0.15; 0.33] at SRT CLTR) of RRMS patients, **17–35%** (CI: [0.70; 0.38] at SRT DR, CI: [0.18; 0.56] at SRT LTS, CI: [0.18; 0.56] at SRT CLTR) of PPMS patients, and **41%–55%** (CI: [0.28; 0.63] at SRT DR, CI: [0.37; 0.72] at SRT LTS, CI: [0.25; 0.60] at SRT CLTR) of SPMS patients are affected. An interesting observation is the impairment of verbal fluency (WLG): in this domain, **57%** CI: [0.36; 0.75] of PPMS patients were affected compared to **45%** CI: [0.28; 0.63] of SPMS, **29%** CI: [0.22; 0.38] of CIS, and **26%** CI: [0.15; 0.42] of RRMS groups. All these prevalence values can be found visually in the summary of Fig 4.

Individual results of each study for each subtype and CDs are detailed in the Supplementary Material, Appendix 7.

#### Further systematic analysis with other cutoff values

Five of the eligible studies could not be included at the meta‐analytic level, because they all used a cutoff value other than the cutoffs of 1.5 SD, 2.0 SD below the normative value or fifth percentile of the normative population to define the DSI, and their modest data were not suitable for statistical analysis of all subtypes; therefore, we conducted only a systematic review of these.

Cutoff values were as follows: at PASAT3 test 1 SD below the mean of healthy controls and a score of 32 correct or less, for the SDMT test a score equal or less than 35 T score and equal or below 55 points, at WLG, SRT‐DR, SRT‐CLTR z score <−1.68.

These studies were largely focused on the RRMS group and were also in line with the previous findings: disregarding the differences in cutoff values, it can be concluded, that IPS impairment affects at least one‐third of RRMS patients.

### Risk of bias assessment and quality of evidence

As most of the included studies were found to be high risk according to at least one, but in most cases two or three aspects of the JBI criteria, in the overall assessment we considered our study as “moderate risk,” which mainly refers to the potential for “selection bias” and “performance bias.”

The assessment of the risk of bias for each included study (listed according to the criteria of the “JBI Quality Assessment Tool for Prevalence Studies”) and ratings of the quality of the evidence are detailed in Supplementary Material, Appendices 8 and 9.

## Discussion

Long overlooked since Jean‐Martin Charcot's first description,^34^ the importance of cognitive symptoms in multiple sclerosis has been brought back into the spotlight over the past 25 years thanks to the explosion of neuropsychological testing, advanced brain imaging techniques and new therapeutic options.

Consequently, the questioning is currently structured around the following aspects: (1) what are the characteristics and patterns, (2) early detection, (3) influencing and predictive factors, and (4) targeted treatment options in MS with CI?^35^

The complexity of this issue and the logical sequence would first require the development of a common professional language at international level: the same definitions, the same NPTs and the same cutoff values should be used to define CI characteristics and prevalence levels, in order to identify predictive factors and to design and implement comparable, targeted therapeutic options. That is, the first step seems to define the characteristics and patterns of CI in MS, along the same assessment criteria.

However, based on the currently available literature, the definition of CI, in particular DSI in MS is not uniform, contradictory and sometimes confusing, both in terms of the tests used and the thresholds for impairment.

For the reasons detailed above, in our meta‐analysis, we selected a prespecified neuropsychological test battery with high specificity and sensitivity, which relatively selectively assesses CDs, and analyzed impairment thresholds to investigate the characteristics and prevalence of cognitive DSI in different clinical subtypes of MS.

In our study, the following general considerations were observed in relation to the results obtained, regardless of the cutoff value used:

CI is observed in all MS phenotypes, with varying degrees, including early forms as well.

Starting from CIS, a broadly tending toward RRMS, PPMS, and finally SPMS, a worsening overall DSI is observed, which basically correlates with the results of previous studies,^19^ although some studies, such as Rosti‐Otajärvi et al. in their analysis found the overall CI was more pronounced in the PPMS group than in SPMS.^36^

Using the BRB‐N test, it was found that the most common cutoffs were 1.5 SD and 2.0 SD below the normal mean and the score below the fifth percentile of the normative values.

Impairment in IPS, referred to in the literature as the “core” symptom, was found to be present in about a quarter of RRMS patients. This is a particularly important finding because, based on the results of EEG, PET, and the most sophisticated functional MRI studies, often used in cognitive psychology, it is now well established that the integrity of cognitive functions is linked to the organization of neuronal networks underlying the ability to process information and attention, that is, impairment to this CD is somehow responsible for the impairment of all other domains (related to DeLuca et al.'s *Relative Consequence Model*, 2004.^37^).^38^

The studies we have included span 20 years and during this period, the therapy of MS has undergone enormous changes. The advent of disease‐modifying therapies (DMT), other new therapeutic approaches (neurorehabilitation, cognitive therapy, dietary approaches, etc.) and multimodal interventions have allowed a demonstrable slowing of disease progression, a significant reduction in relapse rate and long‐term preservation of patients' neuroradiological and physical status (AFFIRM, OPERA I‐II, ASCLEPIOS I‐II, CARE MS I‐II, CLARITY, and CLARITY EXTENSION studies).^39^, ^40^, ^41^, ^42^, ^43^ This primarily begs the question of whether changes in the use of DMTs have had any impact on the prevalence of CI in MS?

Although our study does not directly help to answer this question, given that DMTs have been shown to delay the time of conversion to SPMS (EXPAND, ONTARIO trials)^40^, ^44^ and our study suggests that the SPMS subtype has the highest rates of total DSI, it is conceivable that DMTs may also play a role in rewriting the prevalence rates of DSI. However, more precise analyses are needed to clarify this issue.

Nevertheless, based on the prevalence data obtained in our meta‐analysis, we must see that our results have not provided a consistent clarity to understand the pattern of DSI in MS, which is due to several reasons and gives rise to very important considerations and conclusion.

One of the most important is that the results obtained are obviously influenced by various clinical (physical status‐EDSS score, disease duration, comorbidities, additive affective disorders/mood disorders/depression, sleep disturbances, presence of fatigue, etc.) and sociodemographic (age, gender, race, education, occupational status, marital status, etc.) parameters. For example, a previous cross‐sectional study^19^ examining the prevalence and profile of cognitive dysfunction in different MS subtypes showed that differences in cognitive performance between MS subtypes largely disappeared after controlling for physical disability (EDSS), suggesting that clinical parameters have a crucial impact on cognitive dysfunction in MS.

In our present study, there were insufficient data available for statistical analyses in the included studies in this regard. However, the analyzed patient populations all tend to represent a unique pattern in terms of overall clinical and sociodemographic characteristics, which raises the question of *whether it is even possible to define domain‐specific impairment in a consistent manner, generalized across MS subtypes*. It is also worth mentioning the role of the cognitive reserve as a purely individual characteristic influencing the results, acting as a kind of neuroprotection or compensatory mechanism against the progression of CI and its detectability. The extent and the time of depletion of the cognitive reserve show a high individual variability, although this is occasionally considered in the assessment of cognitive tests (as the “Cognitive Reserve Index”).^35^, ^45^, ^46^

A further reason for the diversity of our results is apparent in the variations on detection of DSI. As mentioned previously, there is and will continue to be a very intense international debate about exactly which CD is being measured by the NPTs that are becoming more widely available. A classic example is the seminal 1991 study by Rao and colleagues describing CI in MS,^9^ which cited verbal fluency as a prominent deficit, but the test measuring this (COWAT) is listed under memory in the article, which probably contributed to the decades‐long disregard of the significance of deficits in verbal fluency in MS.

Furthermore, the cutoff values used to define impairment dichotomously (impaired–not impaired) are also completely inconsistent in the literature. As the definitions of “impairment” and “impaired” vary from study to study—depending on where the cutoff is placed—and this can lead to confusion about nomenclature, it would be preferable to use the term “falling below this cutoff” in scientific comparisons. However, it must be recognized that there is also a sociological and social theoretical dimension to this question, as the perception of what is considered “normal” varies from continent to continent, from country to country, even from region to region. This difference in the definition of reference also implies cultural, subcultural characteristics, making it highly questionable whether *a uniform consensus on the definition of “normative” values can ever be feasibly attained*.

This statement leads us back to the individual evaluation system, in which the reference is the person under examination.

For summary, we should consider whether it is at all possible or desirable to push for a uniform definition of CI in MS, and within that DSI. Instead of a mechanical, definition‐like understanding of DSI, the term *interindividual heterogeneity of cognitive impairment* should be considered in MS, adding, that further studies may be needed in the future to discover trend patterns by comparing very large numbers of individual patterns and extrapolating individual characteristics to the population level (as in the 2021 study by DeMeo et al. in which cognitive phenotype patterns appeared to emerge from comparing individual clinical and neuroradiological parameters^47^).

Additionally, there has been growing recognition that individual variability, including a person's own previous cognitive performance, can be more relevant in assessing cognitive changes in MS. This approach is often referred to as “Reliable Change Indices.”^6^, ^48^, ^49^

Several reasons support the use of *individual referencing* in assessing cognitive achievement in MS:

1. *Baseline variability*: Cognitive function can be influenced by various factors, such as the above‐mentioned clinical és sociodemographic characteritics. Using normative data that does not account for these parameters can lead to misinterpretation. *Individual referencing* allows for a better understanding of how these factors affect a specific patient.
2. *Individual disease course*: MS is a highly variable disease, and cognitive function can change over time. Comparing a patient's cognitive performance to their own previous results allows for a more accurate assessment of disease‐related changes.
3. *Clinical versus lifestyle significance*: What matters most for a patient is whether their cognitive function has changed in a way that is meaningful to their daily life. *Individual referencing* can capture significant clinical and lifestyle changes that are not necessarily reflected in population norms.
4. *Personalized treatment*: Although the 2020 Canadian recommendation^7^ clearly states that optimizing DMTs on the basis of cognitive function is not currently recommended, by monitoring a patient's own cognitive status, clinicians can more effectively adjust neuropsychological interventions and supports to improve quality of life.

In accordance with recommendations from the Consortium of Multiple Sclerosis Centers and the International Multiple Sclerosis Cognition Society,^6^ the Canadian Multiple Sclerosis Working Group (CMSWG)^7^ recommends that a baseline cognitive assessment (with SDMT, „as a minimum”^6^) should be performed in all MS patients at baseline and every 2–3 years (instead of annually^6^ in order to minimize the practice effect) and a 4‐point change or reduction of 10% on SDMT, or change in 0.5 standard deviations, or using Reliable Change Indices is considered to be “clinically meaningful” changes^6^, ^50^ which is equally applicable to *individual referencing* as well.

## Conclusion

Based on our extensive literature review and synthesis of the aggregated evidence, we conclude that, rather than a mechanistic, definition‐bound understanding of cognitive DSI in different subtypes of MS, it is recommended that individuals' own past performance, experiences, and self‐report should be used as a benchmark for cognitive assessment from the moment of the diagnosis. As a key concept, *individual referencing* allows taking into account the individual's unique characteristics and evaluating the results by comparing them to one's own performance. Thus, *individual referencing* means not using a predefined normative population, but using individual data to interpret test results and determine impairment, which obviously requires longitudinal follow‐ups. This would introduce a new approach to cognitive testing that is flexible, personalized, and reflective.

### Strengths and limitations

The strength of our study is that we used the same NPTs and cutoffs to assess different CDIs, which was not the case in previous meta‐analyses on similar topics,^20^, ^51^, ^52^ and we were the first to include CIS patients in the calculations.

Furthermore, it provides a new approach to the topic (*individual referencing*) by evaluating its results.

However, in the absence of sufficient data for further analysis, we have ignored variations in the clinical and sociodemographic parameters underlying the results, which may represent a significant source of bias and limitation in the reporting of results.

In addition, measures of executive function are underrepresented in the BRB‐N battery, meaning that we were unable to perform analyses on this measure.

### Implications for research and clinical practice

By introducing the concept of *individual referencing*, we are pointing in a new direction that may facilitate the assessment of patients' cognitive abilities in clinical work and provide further opportunities for research to make meaningful advances in our knowledge of CI in MS.

## Funding Source

None to declare.

## Author Contributions

**KL:** Conceptualization, acquisition, project administration, formal analysis, and writing—original draft; **MAE:** Conceptualization, project administration, formal analysis, methodology, data curation, and writing—review and editing; **ZH, KH, and DM**: Conceptualization, formal analysis, visualization, and writing—review and editing; **PH**: Conceptualization, funding acquisition, methodology, and writing—review and editing; **PM and GCs**: Conceptualization, data curation, statistics, formal analysis, and writing—review and editing; **ZMo**: Conceptualization and writing—review and editing; **ZMe**: Conceptualization, project administration, formal analysis, data curation, supervision, writing—original draft, and visualization. All authors certify that they have participated sufficiently in the work to take public responsibility for the content, including participation in the concept, design, analysis, writing, or revision of the manuscript.

## Conflict of Interest

KL received speaker fees and conference/travel grants from Biogen, Merck, and Novartis. MAE, ZH, PH, GCs, KH, DM: Nothing to declare. PM received payment as a senior full time biostatistician from the Institute for Translational Medicine, Medical School, University of Pécs. ZMo received payment as a senior medical director from CytoSorbents Europe, Berlin, Germany. ZMe: Received speaker fees and conference/travel grants from: Biogen, Merck, Novartis, Roche, and Sanofi Genzyme.

## Supporting information


Data S1.

Text S1.

## Data Availability

The data underlying this article will be shared on reasonable request to the corresponding author.
