# Supplementary material for: Domain‐specific cognitive impairment in multiple sclerosis: A systematic review and meta‐analysis
Source: Ann Clin Transl Neurol. 2024 Jan 11;11(3):564–76. doi: 10.1002/acn3.51976 (PMC10963281; doi:10.1002/acn3.51976)
Supplement: Supplementary file 1 — Data S1. [file ACN3-11-564-s002.docx]

**SUPPLEMENTARY MATERIAL**

**Katalin Lugosi, Zsolt Mezei et al.: Domain-specific cognitive impairment in multiple sclerosis - A systematic review and meta-analysis**

**Appendix 1.:** PRISMA 2020 Checklist and PRISMA 2020 Abstract Checklist

**Appendix 2.:** Search keys

**Appendix 3.:** Clinical question and CoCoPop framework for literature searching

**Appendix 4.:** Details of the selection and exclusion criteria during the selection process

**Appendix 5.:** List of the references for all included studies

**Appendix 6.,** **eTable 1.:** Baseline characteristics of the included studies

**Appendix 7.:** Individual plots of the domain-specific impairment (DSI) in each MS subtype

**Appendix 8.:** Assessment of risk of bias for each included study (listed per accordance with „JBI Quality Assessment Tool for Prevalence Studies” criteria)

**Appendix 9.:** Ratings of the quality of the evidence

**Appendix 1., PRISMA 2020 Checklist and PRISMA 2020 Abstract Checklist^1^:**

| **Section and Topic** | **Item #** | **Checklist item** | **Location where item is reported** |
| --- | --- | --- | --- |
| **TITLE** | | |  |
| Title | 1 | Identify the report as a systematic review. | Page 1. |
| **ABSTRACT** | | |  |
| Abstract | 2 | See the PRISMA 2020 for Abstracts checklist. | Page 4. |
| **INTRODUCTION** | | |  |
| Rationale | 3 | Describe the rationale for the review in the context of existing knowledge. | Page 5. |
| Objectives | 4 | Provide an explicit statement of the objective(s) or question(s) the review addresses. | Page 6. |
| **METHODS** | | |  |
| Eligibility criteria | 5 | Specify the inclusion and exclusion criteria for the review and how studies were grouped for the syntheses. | Page 6-7., Suppl Mat Appendix 2-4. |
| Information sources | 6 | Specify all databases, registers, websites, organisations, reference lists and other sources searched or consulted to identify studies. Specify the date when each source was last searched or consulted. | Page 6., Suppl Mat Appendix 2., 5. |
| Search strategy | 7 | Present the full search strategies for all databases, registers and websites, including any filters and limits used. | Page 6., Suppl Mat Appendix 2-3. |
| Selection process | 8 | Specify the methods used to decide whether a study met the inclusion criteria of the review, including how many reviewers screened each record and each report retrieved, whether they worked independently, and if applicable, details of automation tools used in the process. | Page 6-7., Figure 1., Suppl Mat Appendix 4. |
| Data collection process | 9 | Specify the methods used to collect data from reports, including how many reviewers collected data from each report, whether they worked independently, any processes for obtaining or confirming data from study investigators, and if applicable, details of automation tools used in the process. | Page 7. |
| Data items | 10a | List and define all outcomes for which data were sought. Specify whether all results that were compatible with each outcome domain in each study were sought (e.g. for all measures, time points, analyses), and if not, the methods used to decide which results to collect. | Page 7-8. |
|  | 10b | List and define all other variables for which data were sought (e.g. participant and intervention characteristics, funding sources). Describe any assumptions made about any missing or unclear information. | Page 7-8. |
| Study risk of bias assessment | 11 | Specify the methods used to assess risk of bias in the included studies, including details of the tool(s) used, how many reviewers assessed each study and whether they worked independently, and if applicable, details of automation tools used in the process. | Page 8. |
| Effect measures | 12 | Specify for each outcome the effect measure(s) (e.g. risk ratio, mean difference) used in the synthesis or presentation of results. | Page 8. |
| Synthesis methods | 13a | Describe the processes used to decide which studies were eligible for each synthesis (e.g. tabulating the study intervention characteristics and comparing against the planned groups for each synthesis (item #5)). | Table 1., Page 7-8., Suppl Mat Appendix 2-4. |
|  | 13b | Describe any methods required to prepare the data for presentation or synthesis, such as handling of missing summary statistics, or data conversions. | Page 7-8. |
|  | 13c | Describe any methods used to tabulate or visually display results of individual studies and syntheses. | Page 7-8., Suppl Mat Appendix 6. |
|  | 13d | Describe any methods used to synthesize results and provide a rationale for the choice(s). If meta-analysis was performed, describe the model(s), method(s) to identify the presence and extent of statistical heterogeneity, and software package(s) used. | Page 7-8. |
|  | 13e | Describe any methods used to explore possible causes of heterogeneity among study results (e.g. subgroup analysis, meta-regression). | Page 7-8. |
|  | 13f | Describe any sensitivity analyses conducted to assess robustness of the synthesized results. | Page 7-8. |
| Reporting bias assessment | 14 | Describe any methods used to assess risk of bias due to missing results in a synthesis (arising from reporting biases). | Page 8. |
| Certainty assessment | 15 | Describe any methods used to assess certainty (or confidence) in the body of evidence for an outcome. | Page 11., Suppl Mat Appendix 8. |
| **RESULTS** | | |  |
| Study selection | 16a | Describe the results of the search and selection process, from the number of records identified in the search to the number of studies included in the review, ideally using a flow diagram. | Page 8-9., Figure 1. |
|  | 16b | Cite studies that might appear to meet the inclusion criteria, but which were excluded, and explain why they were excluded. | Figure 1., Suppl Mat Appendix 4-5. |
| Study characteristics | 17 | Cite each included study and present its characteristics. | Table 1., Suppl Mat Appendix 5. |
| Risk of bias in studies | 18 | Present assessments of risk of bias for each included study. | Page 11., Suppl Mat Appendix 7. |
| Results of individual studies | 19 | For all outcomes, present, for each study: (a) summary statistics for each group (where appropriate) and (b) an effect estimate and its precision (e.g. confidence/credible interval), ideally using structured tables or plots. | Figure 2-4., Suppl Mat Appendix 6. |
| Results of syntheses | 20a | For each synthesis, briefly summarise the characteristics and risk of bias among contributing studies. | Table 1., Suppl Mat Appendix 7. |
|  | 20b | Present results of all statistical syntheses conducted. If meta-analysis was done, present for each the summary estimate and its precision (e.g. confidence/credible interval) and measures of statistical heterogeneity. If comparing groups, describe the direction of the effect. | Page 8-11., Figure 2-4., Suppl Mat Appendix 6. |
|  | 20c | Present results of all investigations of possible causes of heterogeneity among study results. | Page 11-15., Figure 2-4. |
|  | 20d | Present results of all sensitivity analyses conducted to assess the robustness of the synthesized results. | Page 8-11. |
| Reporting biases | 21 | Present assessments of risk of bias due to missing results (arising from reporting biases) for each synthesis assessed. | Page 11., Suppl Mat Appendix 7. |
| Certainty of evidence | 22 | Present assessments of certainty (or confidence) in the body of evidence for each outcome assessed. | Page 11., Suppl Mat Appendix 8. |
| **DISCUSSION** | | |  |
| Discussion | 23a | Provide a general interpretation of the results in the context of other evidence. | Page 11-15. |
|  | 23b | Discuss any limitations of the evidence included in the review. | Page 12. |
|  | 23c | Discuss any limitations of the review processes used. | Page 16. |
|  | 23d | Discuss implications of the results for practice, policy, and future research. | Page 16. |
| **OTHER INFORMATION** | | |  |
| Registration and protocol | 24a | Provide registration information for the review, including register name and registration number, or state that the review was not registered. | Page 6. |
|  | 24b | Indicate where the review protocol can be accessed, or state that a protocol was not prepared. | Page 6. |
|  | 24c | Describe and explain any amendments to information provided at registration or in the protocol. | Page 6. |
| Support | 25 | Describe sources of financial or non-financial support for the review, and the role of the funders or sponsors in the review. | Page 18. |
| Competing interests | 26 | Declare any competing interests of review authors. | Page 17-18. |
| Availability of data, code and other materials | 27 | Report which of the following are publicly available and where they can be found: template data collection forms; data extracted from included studies; data used for all analyses; analytic code; any other materials used in the review. | Page 7-8. |

| **Section and Topic** | **Item #** | **Checklist item** | **Reported (Yes/No)** |
| --- | --- | --- | --- |
| **TITLE** | | |  |
| Title | 1 | Identify the report as a systematic review. | Yes  (Page 1.) |
| **BACKGROUND** | | |  |
| Objectives | 2 | Provide an explicit statement of the main objective(s) or question(s) the review addresses. | Yes (Objective) |
| **METHODS** | | |  |
| Eligibility criteria | 3 | Specify the inclusion and exclusion criteria for the review. | Yes (Methods, 3^rd^-4^th^ sentences |
| Information sources | 4 | Specify the information sources (e.g. databases, registers) used to identify studies and the date when each was last searched. | Yes (Methods, 2^nd^ sentence) |
| Risk of bias | 5 | Specify the methods used to assess risk of bias in the included studies. | Yes (Methods, 6^th^ sentence) |
| Synthesis of results | 6 | Specify the methods used to present and synthesise results. | Yes (Methods, 5^th^ sentence) |
| **RESULTS** | | |  |
| Included studies | 7 | Give the total number of included studies and participants and summarise relevant characteristics of studies. | Yes (Results, 1^st^ sentence) |
| Synthesis of results | 8 | Present results for main outcomes, preferably indicating the number of included studies and participants for each. If meta-analysis was done, report the summary estimate and confidence/credible interval. If comparing groups, indicate the direction of the effect (i.e. which group is favoured). | Yes (Results, 1^st^-2^nd^ sentences) |
| **DISCUSSION** | | |  |
| Limitations of evidence | 9 | Provide a brief summary of the limitations of the evidence included in the review (e.g. study risk of bias, inconsistency and imprecision). | Yes (Interpretation, 2^nd^ sentence) |
| Interpretation | 10 | Provide a general interpretation of the results and important implications. | Yes (Interpretation, 1^st^-2^nd^ sentences) |
| **OTHER** | | |  |
| Funding | 11 | Specify the primary source of funding for the review. | No funding |
| Registration | 12 | Provide the register name and registration number. | Yes (Methods, 1^st^ sentence) |

**Appendix 2., Search keys:**

**MEDLINE (via PubMed):**

„multiple sclerosis” AND (cognitive OR cognition OR neurocognitive OR neurocognition) AND (impairment OR decline OR dysfunction)

**Cochrane Library (CENTRAL):**

„multiple sclerosis” AND (cognitive OR cognition OR neurocognitive OR neurocognition) AND (impairment OR decline OR dysfunction)

**Embase:**

„multiple sclerosis” AND (cognitive OR cognition OR neurocognitive OR neurocognition) AND (impairment OR decline OR dysfunction)

**Appendix 3., Clinical question and CoCoPop framework for literature searching:**

**Q:** What is the prevalence of domain-specific impairment (DSI) in different clinical subtypes of multiple sclerosis (MS) based on the Brief Repetable Battery of Neuropsychological Tests?

**Co (condition):** distinct DSI measured by BRB-N (Brief Repeatable Battery of Neuropsychological Tests) composit test series which includes: PASAT-3 (paced auditory serial addition test-3), SDMT (symbol digit modalities test), WLG (word list generation test – phonetic fluency and verbal fluency), 10/36-spatial recall test (immediate recall, long-term storage, delayed recall), SRT (Buschke’s selective reminding test: long-term storage, consistent long-term retrieval, delayed recall) tests

**Co (context):** different clinical subtypes of MS (clinically isolated syndrome/CIS, relapsing-remitting/RRMS, primary progressive/PPMS, secondary progressive/SPMS) according to Lublin classification

**Pop (population):** adult patients (≥18 years) of both sexes diagnosed with MS

**Appendix 4., Details of the selection and exclusion criteria during the selection process:**

**Inclusion criteria:**

- MS diagnosis based on the McDonald Criteria (2001^2^, 2005^3^, 2010^4^, 2017^5^ McDonald Criteria for the Diagnosis of Multiple Sclerosis)
- If it is not stated which diagnostic criteria was used, but the study was generated after 2001
- Study designs: Clinical observational studies (i.e. retrospective cohort studies, prospective cohort studies, case-control studies, longitudinal or follow-up studies providing baseline cross-sectional data, cross-sectional studies), accepted manuscripts
- Adult MS patients (≥18years) of both sexes with a precisely defined MS subtype (i.e. CIS=clinically isolated syndrome, RRMS=relapsing-remitting MS, PPMS=primary progressive MS, SPMS=secondary progressive MS)
- At least one of the BRB-N subtests (PASAT3: Paced Auditory Serial Addition Test 3), SDMT (Symbol Digit Modalities Test), WLG (Word List Generation test – phonetic fluency and verbal fluency), 10/36-SPART (10/36 SPAtial Recall Test – IR: immediate recall, LTS: long-term storage, DR: delayed recall), SRT (Selective Reminding Rest – LTS: long-term storage, CLTR: consistent long-term retrieval, DR: delayed recall) tests
- Testing ≥1month after relapse/shub/steroid administration
- Measurements with the relevant subtests of the following composit test series corresponding to the BRB-N: MACFIMS (Minimal Assessment of Cognitive Function in MS; SDMT, PASAT 3 included), BICAMS (Brief International Cognitive Assessment for MS; SDMT included), NSBMS (Neuropsychological Screening Battery for Multiple Sclerosis; PASAT 3, SRT, WLG included) and MSFC (Multiple Sclerosis Functional Composite; PASAT 3 included)
- Studies with age-, education-, disease stage-, disease duration- restrictions

**Exclusion criteria:**

- The use of all diagnostic criteria other than the McDonald for the diagnosis of MS
- Studies before 2001 (since McDonald Criteria was first established in 2001, studies published at least before 2001 were excluded)
- Study designs: RCTs (randomized controlled trials), review, case series, case report, conference abstracts, poster sessions, e-presentation sessions, poster abstracts, corrigendums, conference posters, conference supplements, book chapter
- Pediatric population or „pediatric-onset multiple sclerosis (POMS)”
- Computerized or electronic version of the subtests of BRB-N (clinically meaningful differences)
- Measuring with 7/24 SPART version of the SPART test
- Testing during relapse/shub/steroid administration
- PASAT 2 or generally mentioned “PASAT” and “digit symbol test”
- RIS: radiologically isolated syndrome
- not MS-related topic
- only healthy populations were tested
- non-neurological disease was investigated
- no data are available on the prevalence rate of impaired patients in the BRB-N subtests
- gender restriction (e.g. only females were investigated)
- MS patients not specified for a subtype
- MS patients or MS subtypes not specified for a subtests of BRB-N (e.g. „overall cognitive impairment”)

Notes:

- During the title&abstract selection, we included studies generally mentioning „patient with multiple sclerosis (PwMS)” or „multiple sclerosis” (in many cases only the full-text contains the exact MS subtype)

- During the title&abstract selection, we included studies generally mentioning “neuropsychological assessment”, “cognitive testing”, “the attention functions, memory, language, visuo-spatial abilities, and executive functions were evaluated/assessed”; “various cognitive tests”; “the subjects underwent a neuropsychometric test battery to evaluate cognitive functions, including memory, visuospatial, and executive functions”; “administered a battery of neuropsychological tests”, “patients underwent neuropsychological tests” etc. (in many cases only the full-text contains the exact measurements)

- In case of a study presenting data from overlapping or completely identical/overlap populations, we have chosen the one with the best quality regarding the available data

- For one study^6^, 2 cut-off values were included in the statistical analyses (in the meta-analysis section), as it used 2 different cut-off values to define cognitive domain impairment

- There were no language restrictions

- A study was considered acceptable even if its primary objective was not to determine the prevalence value (e.g. MRI studies)

**Appendix 5.: List of the references for all included studies:**

1. Altieri M, Fratino M, Maestrini I et al. Cognitive Performance in Relapsing-Remitting Multiple Sclerosis: At Risk or Impaired? Dement Geriatr Cogn Disord. 2020;49(6):539-543. doi: 10.1159/000514674. Epub 2021 Mar 18. PMID: 33735893.
2. Amato MP, Portaccio E, Goretti B et al. Association of neocortical volume changes with cognitive deterioration in relapsing-remitting multiple sclerosis. Arch Neurol. 2007 Aug;64(8):1157-61. doi: 10.1001/archneur.64.8.1157. PMID: 17698706.
3. Amato MP, Razzolini L, Goretti B et al. Cognitive reserve and cortical atrophy in multiple sclerosis: a longitudinal study. Neurology. 2013 May 7;80(19):1728-33. doi: 10.1212/WNL.0b013e3182918c6f. Epub 2013 Apr 10. PMID: 23576622.
4. Artemiadis A, Anagnostouli M, Zalonis I, Chairopoulos K, Triantafyllou N. Structural MRI correlates of cognitive function in multiple sclerosis. Mult Scler Relat Disord. 2018 Apr;21:1-8. doi: 10.1016/j.msard.2018.02.003. Epub 2018 Feb 6. PMID: 29438835.
5. Berrigan LI, Lefevre JA, Rees LM, Berard J, Freedman MS, Walker LA. Cognition in early relapsing-remitting multiple sclerosis: consequences may be relative to working memory. J Int Neuropsychol Soc. 2013 Sep;19(8):938-49. doi: 10.1017/S1355617713000696. Epub 2013 Jul 18. PMID: 23866100.
6. Bisecco A, Rocca MA, Pagani E et al.; MAGNIMS Network. Connectivity-based parcellation of the thalamus in multiple sclerosis and its implications for cognitive impairment: A multicenter study. Hum Brain Mapp. 2015 Jul;36(7):2809-25. doi: 10.1002/hbm.22809. Epub 2015 Apr 14. PMID: 25873194; PMCID: PMC6869750.
7. Caceres F, Vanotti S, Benedict RH; RELACCEM Work Group. Cognitive and neuropsychiatric disorders among multiple sclerosis patients from Latin America: Results of the RELACCEM study. Mult Scler Relat Disord. 2014 May;3(3):335-40. doi: 10.1016/j.msard.2013.10.007. Epub 2013 Nov 6. PMID: 25876470.
8. Calabrese M, Agosta F, Rinaldi F et al. Cortical lesions and atrophy associated with cognitive impairment in relapsing-remitting multiple sclerosis. Arch Neurol. 2009 Sep;66(9):1144-50. doi: 10.1001/archneurol.2009.174. PMID: 19752305.
9. Clough M, Dobbing J, Stankovich J et al. Cognitive processing speed deficits in multiple sclerosis: Dissociating sensorial and motor processing changes from cognitive processing speed. Mult Scler Relat Disord. 2020 Feb;38:101522. doi: 10.1016/j.msard.2019.101522. Epub 2019 Nov 22. PMID: 31785491.
10. Dackovic J, Pekmezovic T, Mesaros S et al. The Rao's Brief Repeatable Battery in the study of cognition in different multiple sclerosis phenotypes: application of normative data in a Serbian population. Neurol Sci. 2016 Sep;37(9):1475-81. doi: 10.1007/s10072-016-2610-1. Epub 2016 May 20. PMID: 27207679.
11. Davion JB, Lopes R, Jougleux C et al. Brief International Cognitive Assessment for Multiple Sclerosis scores are associated with the cortical thickness of specific cortical areas in relapsing-remitting patients. Rev Neurol (Paris). 2022 Apr;178(4):326-336. doi: 10.1016/j.neurol.2021.06.014. Epub 2021 Oct 14. PMID: 34657733.
12. Eilam-Stock T, Shaw MT, Krupp LB, Charvet LE. Early neuropsychological markers of cognitive involvement in multiple sclerosis. J Neurol Sci. 2021 Apr 15;423:117349. doi: 10.1016/j.jns.2021.117349. Epub 2021 Feb 17. PMID: 33639421.
13. Fenu G, Arru M, Lorefice L et al. Does focal inflammation have an impact on cognition in multiple sclerosis? An MRI study. Mult Scler Relat Disord. 2018 Jul;23:83-87. doi: 10.1016/j.msard.2018.05.012. Epub 2018 May 16. PMID: 29800885.
14. Feuillet L, Reuter F, Audoin B et al. Early cognitive impairment in patients with clinically isolated syndrome suggestive of multiple sclerosis. Mult Scler. 2007 Jan;13(1):124-7. doi: 10.1177/1352458506071196. PMID: 17294621.
15. Forn C, Rocca MA, Valsasina P et al. Functional magnetic resonance imaging correlates of cognitive performance in patients with a clinically isolated syndrome suggestive of multiple sclerosis at presentation: an activation and connectivity study. Mult Scler. 2012 Feb;18(2):153-63. doi: 10.1177/1352458511417744. Epub 2011 Aug 9. PMID: 21828200.
16. Hegedüs K, Kárpáti J, Iljicsov A, Simó M. Neuropsychological characteristics of benign multiple sclerosis patients: A two-year matched cohort study. Mult Scler Relat Disord. 2019 Oct;35:150-155. doi: 10.1016/j.msard.2019.07.022. Epub 2019 Jul 27. PMID: 31376686.
17. Iancheva D, Trenova A, Mantarova S, Terziyski K. Functional Magnetic Resonance Imaging Correlations Between Fatigue and Cognitive Performance in Patients With Relapsing Remitting Multiple Sclerosis. Front Psychiatry. 2019 Oct 29;10:754. doi: 10.3389/fpsyt.2019.00754. PMID: 31749716; PMCID: PMC6842936.
18. Labiano-Fontcuberta A, Martínez-Ginés ML, Aladro Y et al. A comparison study of cognitive deficits in radiologically and clinically isolated syndromes. Mult Scler. 2016 Feb;22(2):250-3. doi: 10.1177/1352458515591072. Epub 2015 Jun 17. PMID: 26084350.
19. Laffon M, Malandain G, Joly H, Cohen M, Lebrun C. The HV3 Score: A New Simple Tool to Suspect Cognitive Impairment in Multiple Sclerosis in Clinical Practice. Neurol Ther. 2014 Nov 25;3(2):113-22. doi: 10.1007/s40120-014-0021-x. PMID: 26000227; PMCID: PMC4386426.
20. Lebrun C, Blanc F, Brassat D, Zephir H, de Seze J; CFSEP. Cognitive function in radiologically isolated syndrome. Mult Scler. 2010 Aug;16(8):919-25. doi: 10.1177/1352458510375707. Epub 2010 Jul 7. PMID: 20610492.
21. Loitfelder M, Fazekas F, Koschutnig K et al. Brain activity changes in cognitive networks in relapsing-remitting multiple sclerosis - insights from a longitudinal FMRI study. PLoS One. 2014 Apr 9;9(4):e93715. doi: 10.1371/journal.pone.0093715. PMID: 24718105; PMCID: PMC3981758.
22. Lozano-Soto E, Cruz-López ÁJ, Gutiérrez R et al. Predicting Neuropsychological Impairment in Relapsing Remitting Multiple Sclerosis: The Role of Clinical Measures, Treatment, and Neuropsychiatry Symptoms. Arch Clin Neuropsychol. 2021 May 21;36(4):475-484. doi: 10.1093/arclin/acaa088. Erratum in: Arch Clin Neuropsychol. 2021 Aug 31;36(6):1019. PMID: 33067616.
23. Mainero C, Caramia F, Pozzilli C et al. fMRI evidence of brain reorganization during attention and memory tasks in multiple sclerosis. Neuroimage. 2004 Mar;21(3):858-67. doi: 10.1016/j.neuroimage.2003.10.004. PMID: 15006652.
24. Marstrand L, Østerberg O, Walsted T, Skov AC, Schreiber KI, Sellebjerg F. Brief international cognitive assessment for multiple sclerosis (BICAMS): A danish validation study of sensitivity in early stages of MS. Mult Scler Relat Disord. 2020 Jan;37:101458. doi: 10.1016/j.msard.2019.101458. Epub 2019 Oct 18. Erratum in: Mult Scler Relat Disord. 2020 Feb;38:101942. PMID: 31683230.
25. Mashayekhi F, Sadigh-Eteghad S, Naseri A, Asadi M, Abbasi Garravnd N, Talebi M. ApoE4-positive multiple sclerosis patients are more likely to have cognitive impairment: a cross-sectional study. Neurol Sci. 2022 Feb;43(2):1189-1196. doi: 10.1007/s10072-021-05383-z. Epub 2021 Jun 12. PMID: 34120271.
26. Maubeuge N, Deloire MSA, Brochet B et al.; BICAFMS study investigators. Validation of the French version of the minimal assessment of cognitive function in multiple sclerosis (MACFIMS). Mult Scler Relat Disord. 2021 Feb;48:102692. doi: 10.1016/j.msard.2020.102692. Epub 2020 Dec 17. PMID: 33352358.
27. Megna R, Alfano B, Lanzillo R et al. Brain tissue volumes and relaxation rates in multiple sclerosis: implications for cognitive impairment. J Neurol. 2019 Feb;266(2):361-368. doi: 10.1007/s00415-018-9139-6. Epub 2018 Nov 29. PMID: 30498912.
28. Migliore S, Ghazaryan A, Simonelli I et al. Cognitive Impairment in Relapsing-Remitting Multiple Sclerosis Patients with Very Mild Clinical Disability. Behav Neurol. 2017;2017:7404289. doi: 10.1155/2017/7404289. Epub 2017 Aug 15. PMID: 28912625; PMCID: PMC5574272.
29. Moccia M, Lanzillo R, Palladino R et al. Cognitive impairment at diagnosis predicts 10-year multiple sclerosis progression. Mult Scler. 2016 Apr;22(5):659-67. doi: 10.1177/1352458515599075. Epub 2015 Sep 11. PMID: 26362896.
30. Moccia M, Lanzillo R, Costabile T et al. Uric acid in relapsing-remitting multiple sclerosis: a 2-year longitudinal study. J Neurol. 2015;262(4):961-7. doi: 10.1007/s00415-015-7666-y. Epub 2015 Feb 12. PMID: 25673130.
31. Moroso A, Ruet A, Lamargue-Hamel D et al. Posterior lobules of the cerebellum and information processing speed at various stages of multiple sclerosis. J Neurol Neurosurg Psychiatry. 2017 Feb;88(2):146-151. doi: 10.1136/jnnp-2016-313867. Epub 2016 Oct 27. PMID: 27789541.
32. Nogales-Gaete J, Aracena R, Díaz V et al. Evaluación neuropsicológica en 129 pacientes chilenos con esclerosis múltiple recurrente remitente previo a inicio de fármacos inmunomoduladores [Neuropsychological assessment of patients with relapsing remitting multiple sclerosis prior to the use of immunomodulatory drugs]. Rev Med Chil. 2012 Nov;140(11):1437-44. Spanish. doi: 10.4067/S0034-98872012001100009. PMID: 23677190.
33. Ntoskou K, Messinis L, Nasios G et al. Cognitive and Language Deficits in Multiple Sclerosis: Comparison of Relapsing Remitting and Secondary Progressive Subtypes. Open Neurol J. 2018 Mar 12;12:19-30. doi: 10.2174/1874205X01812010019. PMID: 29576812; PMCID: PMC5850485.
34. Ozakbas S, Yigit P, Cinar BP, Limoncu H, Kahraman T, Kösehasanoğulları G. The Turkish validation of the Brief International Cognitive Assessment for Multiple Sclerosis (BICAMS) battery. BMC Neurol. 2017 Dec 6;17(1):208. doi: 10.1186/s12883-017-0993-0. PMID: 29207954; PMCID: PMC5717821.
35. Ozturk A, Smith SA, Gordon-Lipkin EM et al. MRI of the corpus callosum in multiple sclerosis: association with disability. Mult Scler. 2010 Feb;16(2):166-77. doi: 10.1177/1352458509353649. PMID: 20142309; PMCID: PMC2820126.
36. Pavisian B, Patel VP, Feinstein A. Cognitive mediated eye movements during the SDMT reveal the challenges with processing speed faced by people with MS. BMC Neurol. 2019 Dec 26;19(1):340. doi: 10.1186/s12883-019-1543-8. PMID: 31878918; PMCID: PMC6933713.
37. Planche V, Ruet A, Coupé P et al. Hippocampal microstructural damage correlates with memory impairment in clinically isolated syndrome suggestive of multiple sclerosis. Mult Scler. 2017 Aug;23(9):1214-1224. doi: 10.1177/1352458516675750. Epub 2016 Oct 25. PMID: 27780913.
38. Pokryszko-Dragan A, Dziadkowiak E, Zagrajek M et al. Cognitive performance, fatigue and event-related potentials in patients with clinically isolated syndrome. Clin Neurol Neurosurg. 2016 Oct;149:68-74. doi: 10.1016/j.clineuro.2016.07.022. Epub 2016 Jul 15. PMID: 27484631.
39. Portaccio E, Goretti B, Zipoli V et al. APOE-epsilon4 is not associated with cognitive impairment in relapsing-remitting multiple sclerosis. Mult Scler. 2009 Dec;15(12):1489-94. doi: 10.1177/1352458509348512. Epub 2009 Nov 13. PMID: 19965518.
40. Potagas C, Giogkaraki E, Koutsis G et al. Cognitive impairment in different MS subtypes and clinically isolated syndromes. J Neurol Sci. 2008 Apr 15;267(1-2):100-6. doi: 10.1016/j.jns.2007.10.002. Epub 2007 Nov 13. PMID: 17997417.
41. Reuter F, Zaaraoui W, Crespy L et al. Cognitive impairment at the onset of multiple sclerosis: relationship to lesion location. Mult Scler. 2011 Jun;17(6):755-8. doi: 10.1177/1352458511398265. Epub 2011 Mar 3. PMID: 21372116.
42. Riccitelli G, Rocca MA, Pagani E et al. Cognitive impairment in multiple sclerosis is associated to different patterns of gray matter atrophy according to clinical phenotype. Hum Brain Mapp. 2011 Oct;32(10):1535-43. doi: 10.1002/hbm.21125. Epub 2010 Aug 25. PMID: 20740643; PMCID: PMC6870304.
43. Rocca MA, Riccitelli G, Rodegher M et al. Functional MR imaging correlates of neuropsychological impairment in primary-progressive multiple sclerosis. AJNR Am J Neuroradiol. 2010 Aug;31(7):1240-6. doi: 10.3174/ajnr.A2071. Epub 2010 Mar 18. PMID: 20299439; PMCID: PMC7965463.
44. Sandi D, Rudisch T, Füvesi J et al. Hungarian validation of the Brief International Cognitive Assessment for Multiple Sclerosis (BICAMS) battery and the correlation of cognitive impairment with fatigue and quality of life. Mult Scler Relat Disord. 2015 Nov;4(6):499-504. doi: 10.1016/j.msard.2015.07.006. Epub 2015 Jul 8. PMID: 26590654.
45. Scherer P, Baum K, Bauer H, Göhler H, Miltenburger C. Normierung der Brief Repeatable Battery of Neuropsychological Tests (BRB-N) für den deutschsprachigen Raum Anwendung bei schubförmig remittierenden und sekundär progredienten Multiple-Sklerose-Patienten [Normalization of the Brief Repeatable Battery of Neuropsychological tests (BRB-N) for German-speaking regions. Application in relapsing-remitting and secondary progressive multiple sclerosis patients]. Nervenarzt. 2004 Oct;75(10):984-90. German. doi: 10.1007/s00115-004-1729-0. PMID: 15118827.
46. Skorve E, Lundervold AJ, Torkildsen Ø, Myhr KM. The Norwegian translation of the brief international cognitive assessment for multiple sclerosis (BICAMS). Mult Scler Relat Disord. 2019 Nov;36:101408. doi: 10.1016/j.msard.2019.101408. Epub 2019 Sep 21. PMID: 31610403.
47. Vanotti S, Cores EV, Eizaguirre B, Melamud L, Rey R, Villa A. Cognitive performance of neuromyelitis optica patients: comparison with multiple sclerosis. Arq Neuropsiquiatr. 2013 Jun;71(6):357-61. doi: 10.1590/0004-282X20130038. PMID: 23828523.
48. Viterbo RG, Iaffaldano P, Trojano M. Verbal fluency deficits in clinically isolated syndrome suggestive of multiple sclerosis. J Neurol Sci. 2013 Jul 15;330(1-2):56-60. doi: 10.1016/j.jns.2013.04.004. Epub 2013 Apr 28. PMID: 23628466.

**Appendix 6.: eTable 1. Baseline characteristics of the included studies:**

| **Study, year** | **Country** | **Number of patients** | **Number of females (%)** | **Age^b^** | **Used tests** | **Subtype(s) investigated** | **Cut-off** |
| --- | --- | --- | --- | --- | --- | --- | --- |
| Feuillet et al., 2007 | France | 40 | 32 (80%) | 30.9±6.7 | SDMT, PASAT3, WLG, SRT, SPART | CIS | 2.0 SD below the normative value |
| Labiano-Fontcuberta et al., 2015 | Spain | 25 | 19 (76%) | 39.8±6 | SDMT, PASAT3, WLG, SRT-DR, SRT-LTS, SRT-CLTR, SPART, SPART-DR |  |  |
| Viterbo et al., 2013 | Italy | 100 | 59 (59%) | nd | SDMT, PASAT3, WLG, SRT-DR, SRT-LTS, SRT-CLTR, SPART, SPART-DR |  |  |
| Altieri et al., 2020 | Italy | 82 | 56 (68%) | 45.8±11 | SDMT | RRMS |  |
| Amato et al., 2007 | Italy | 41 | nd | nd | SDMT, PASAT3, WLG, SRT-DR, SRT-LTS, SRT-CLTR, SPART, SPART-DR |  |  |
| Bisecco et al., 2015 | Netherlands, Austria, UK, Italy | 52 | 33 (63%) | 40.3±8.5 | SRT, WLG, SPART |  |  |
| Calabrese et al., 2009 | Italy | 70 | 45 (64%) | 34.8 | SPART |  |  |
| Laffon et al., 2014 | France | 75 | 57 (76%) | 36.1±9.2 | PASAT-3 |  |  |
| Lebrun et al., 2010 | France | 26 | 23 (88%) | 39.61±12.1 | PASAT-3, WLG, SRT, SPART, SPART-DR |  |  |
| Loitfelder et al., 2014 | Austria | 15 | nd | nd | SDMT, PASAT3, WLG, SRT-LTS, SRT-CLTR, SPART, SPART-DR |  |  |
| Mainero et al., 2004 | Italy | 22 | 14 (64%) | 30.5 (median) | SDMT, WLG, SRT, SPART |  |  |
| Megna et al., 2019 | Italy | 186 | nd | nd | SDMT, PASAT3, WLG, SRT-DR, SRT-LTS, SRT-CLTR, SPART, SPART-DR |  |  |

| **eTable 1. Baseline characteristics of the included studies *(continued)*:** | | | | | | | |
| --- | --- | --- | --- | --- | --- | --- | --- |
| **Study, year** | **Country** | **Number of patients** | **Number of females (%)** | **Age^b^** | **Used tests** | **Subtype(s) investigated** | **Cut-off** |
| Moccia et al., 2015 (A) | Italy | 155 | 99 (64%) | 32.1±8.5 | SDMT, PASAT3, WLG, SRT-DR, SRT, SRT-CLTR, SPART, SPART-DR | RRMS | 2.0 SD below the normative value |
| Moccia et al., 2015 | Italy | 141 | 81 (57%) | 35.7±8.2 | SDMT |  |  |
| Nogales-Gaete et al., 2012^c^ | Chile | 129 | 89 (69%) | 33.4±9.5 | SDMT, PASAT3, SRT-DR, SRT-LTS, SRT-CLTR |  |  |
| Portaccio et al., 2009 | Italy | 85 | 58 (68%) | 43±8.4 | SDMT, PASAT3, WLG, SRT-DR, SRT-LTS, SRT-CLTR, SPART, SPART-DR |  |  |
| Rocca et al., 2010^e^ | Italy | 16 | 9 (56%) PPMS | 49.7, range: 39-68 | PASAT-3 | PPMS |  |
| Riccitelli et al., 2010 | Italy | 22 RRMS  22 PPMS  29 SPMS | 17 (77%) RRMS  17 (77%) RRMS  20 (69%) SPMS | 40.9, range: 22-63 (RRMS)  40.9, range: 22-63 (PPMS)  45.4, range: 27-61 (SPMS) | PASAT-3 | RRMS  PPMS  SPMS |  |
| Moroso et al., 2016 | France | 37 | 29 (78%) | 36 (median) 19-59 (range) | SDMT | CIS | 1.5 SD below the normative value  1.5 SD below the normative value |
| Planche et al., 2016 | France | 37 | 29 (78%) | 37.4, range: 19-59 | SDMT |  |  |
| Pokryszko-Dragan et al., 2016 | Poland | 44 | 27 (61%) | 31.4, range: 21-48 | SDMT, PASAT3, WLG, SRT-DR, SRT-LTS, SRT-CLTR, SPART, SPART-DR |  |  |
| Berrigan et al., 2013 | Canada | 70 | 57 (81%) | 40.34±8.78 | SDMT, PASAT3 | RRMS  RRMS |  |
| Clough et al., 2020 | Australia | 22 | 18 (82%) | 47.4±8.73 | SDMT |  |  |
| Davion et al., 2021 | France | 96 | 70 (73%) | 40.7±11.7 (median±SD) | SDMT |  |  |
| Eilam-Stock et al., 2021 | USA | 25 | 14 (56%) | 26.15±5.18 | SDMT |  |  |
| Hegedüs et al., 2019 | Hungary | 22 | 17 (77%) | 45.1±9.2 | PASAT-3 |  |  |
| Lozano-Soto et al., 2020 | Spain | 91 | 63 (69%) | 48.6±8.8 | SDMT, PASAT3 |  |  |
| Marstrand et al., 2019 | Denmark | 65 | 41 (63%) | 37.2±8.8 | SDMT |  |  |
| Mashayekhi et al., 2020 | Iran | 71 | 53 (75%) | 31.43±8.75 | SDMT, PASAT3 |  |  |
| Pavisian et al., 2019 | Canada | 33 | 22 (70%) | 41.42±9.89 | SDMT |  |  |
| Sandi et al., 2015^f^ | Hungary | 65 | 49 (75%) | 41.9±8.9 | SDMT |  |  |
| Skorve et al., 2019 | Norway | 65 | 42 (65%) | 37.02±10.4 | SDMT |  |  |
| Dackovic et al., 2016 | Serbia | 37 CIS  65 RRMS  35 PPMS  31 SPMS | 21 (57%) CIS  45 (69%) RRMS  20 (57%) PPMS  21 (68%) SPMS | 30.6±7.7 (CIS)  37.8±11 (RRMS)  46.8±10.1 (PPMS)  46.8±9.1 (SPMS) | SDMT, PASAT3, WLG, SRT-DR, SRT-LTS, SRT-CLTR, SPART, SPART-DR | CIS  RRMS  PPMS  SPMS |  |
| Maubeuge et al., 2021 | France | 32 RRMS  34 PPMS  35 SPMS | nd | nd | SDMT, PASAT3 | RRMS  PPMS  SPMS |  |
| Ntoskou et al., 2018 | Greece | 15 RRMS  12 SPMS | 12 (80%) RRMS  9 (75%) SPMS | 43.6±9.74 (RRMS), 48.67±8.07 (SPMS) | SDMT | RRMS  SPMS |  |
| Reuter et al., 2011 | France | 97 | 77 (79%) | 31±8 | SDMT, PASAT3, WLG, SRT-DR, SRT-LTS, SRT-CLTR, SPART, SPART-DR | CIS | 5th percentile below the normative value  5th percentile below the normative value |
| Amato et al., 2013 | Italy | 52 | 37 (71%) | 39.2±9 | SDMT, SRT, PASAT3, SPART | RRMS  RRMS |  |
| Artemiadis et al., 2018 | Greece | 61 | 44 (72%) | 41.8±10.6 | SDMT |  |  |
| Caceres et al., 2014 | Latin America: Argentina Chile Columbia Venezuela Uruguay and Mexico | 110 | 74 (67%) | 36.6±10.6 | SDMT, PASAT3, WLG, SRT-DR, SRT-LTS |  |  |
| Vanotti et al., 2013 | Argentina | 14 | nd | 37.93±10.57 | SRT |  |  |
| Ozakbas et al., 2017 | Turkey | 151 RRMS  4 PPMS  18 SPMS | nd | nd | SDMT | RRMS  PPMS  SPMS |  |
| Potagas et al., 2008 | Greece | 33 CIS  75 RRMS  23 PPMS  29 SPMS | 18 (55%) CIS  51 (68%) RRMS  13 (57%) PPMS  16 (55%) SPMS | 34.7±8.7 (CIS)  34.3±8.9 (RRMS)  42.8±9.9 (PPMS)  42.0±8.5 (SPMS) | SDMT, PASAT3, WLG, SRT-DR, SRT-LTS, SRT-CLTR, SPART, SPART-DR | CIS  RRMS  PPMS  SPMS |  |
| Migliore et al., 2017 | Italy | 92 | 64 (70%) | 41.5±10.7 | SDMT, PASAT3 | RRMS | 5th percentile below the normative value  and 1.5 SD below the normative value^h^ |
| Fenu et al., 2018^a^ | Italy | 84 | 65 (77%) | 42.12±10.89 | SDMT | RRMS | ≤35 T score |
| Forn et al., 2011^a,g^ | Spain | 18 | nd | 32.9 | PASAT3 | CIS | 1.0 SD below the mean of HC |
| Iancheva et al., 2019^a^ | Bulgaria | 42 | nd | nd | SDMT | RRMS | ≤55points |
| Ozturk et al., 2010^a^ | USA | 28 RRMS  12 PPMS  13 SPMS | 18 (64%) RRMS  8 (67%) PPMS  7 (54%) SPMS | 40 (RRMS) (median)  53 (PPMS) (median)  52 (SPMS) (median) | PASAT-3 | RRMS  PPMS  SPMS | ≤32 correct points |
| Sherer et al., 2004^a,d^ | Germany | 43 RRMS  60 SPMS | 31 (73%) RRMS  40 (67%) SPMS | 34, range: 18-55 (RRMS)  42.7, range: 27-56 (SPMS) | WLG, SRT-DR, SRT-CLTR | RRMS  SPMS | z≤-1.68 |

^a^a systematic review was conducted

^b^age expressed as mean±SD (in years), unless otherwise indicated

^c^written in Spanish language

^d^written in German language

^e^a cut-off value based on a reference of the study

^f^a cut-off value based on the consultation with the authors

^g^as individual age data were provided, the average was calculated on this basis

^h^data based on two cut-off values

nd: no data

HC: healthy controls

(A): a non-overlapping study population from the same author, dating from the same year

**Appendix 7., Individual plots of the domain-sepcific impairment (DSI) in each MS subtype (the subtypes and the cognitive domains tested, as well as the tests used to measure them, are indicated below the figures):**

**
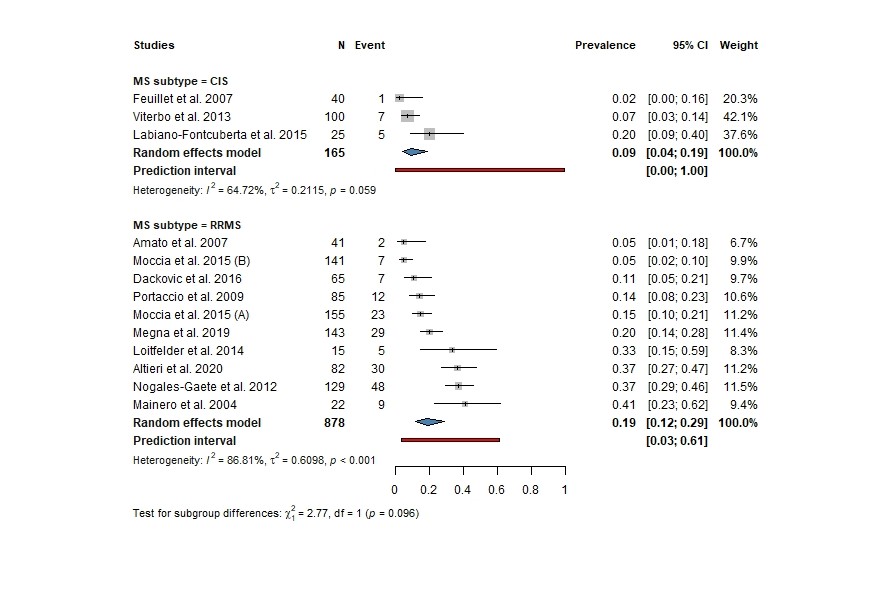
**

**eFigure 1.** Individual plot of information processing speed (IPS)/attention DSI (domain-specific impairment) prevalence rates measured by Symbol Digit Modalities Test (SDMT) at cut-off of 2.0 SD below the normative value.

**
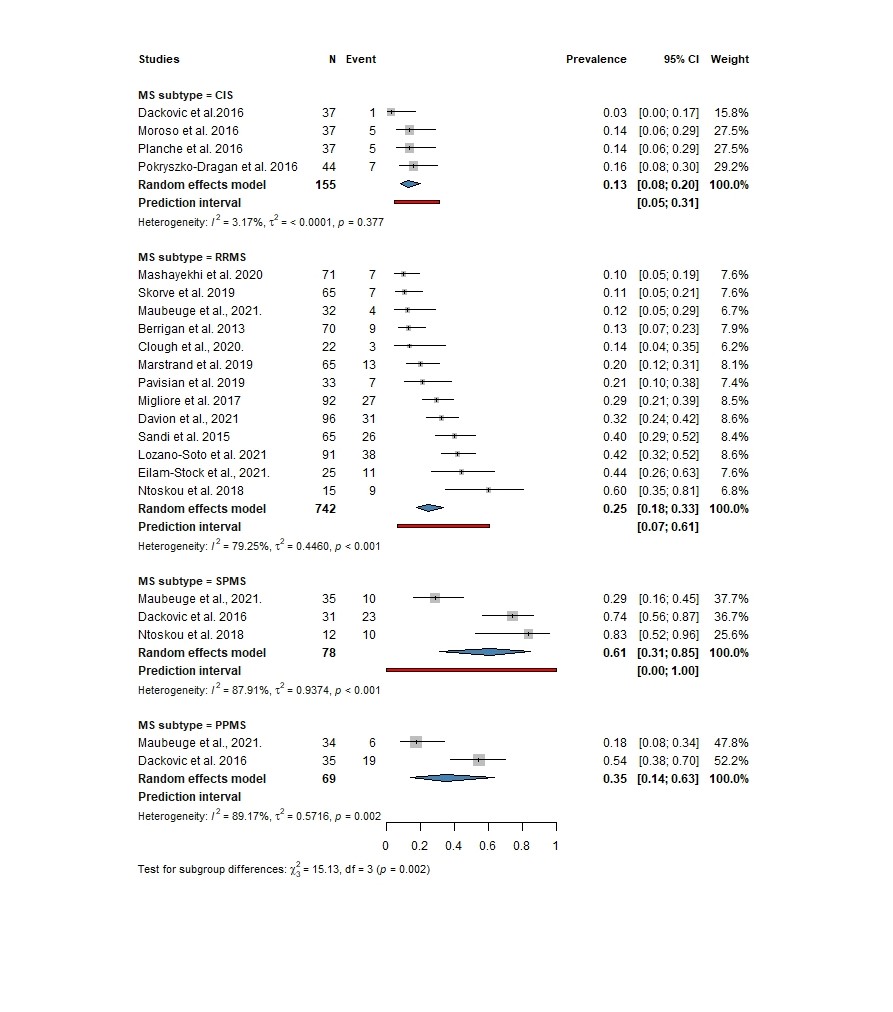
**

**eFigure 2.** Individual plot of information processing speed (IPS)/attention DSI (domain-specific impairment) prevalence rates measured by Symbol Digit Modalities Test (SDMT) at the cut-off of 1.5 SD below the normative value.

**
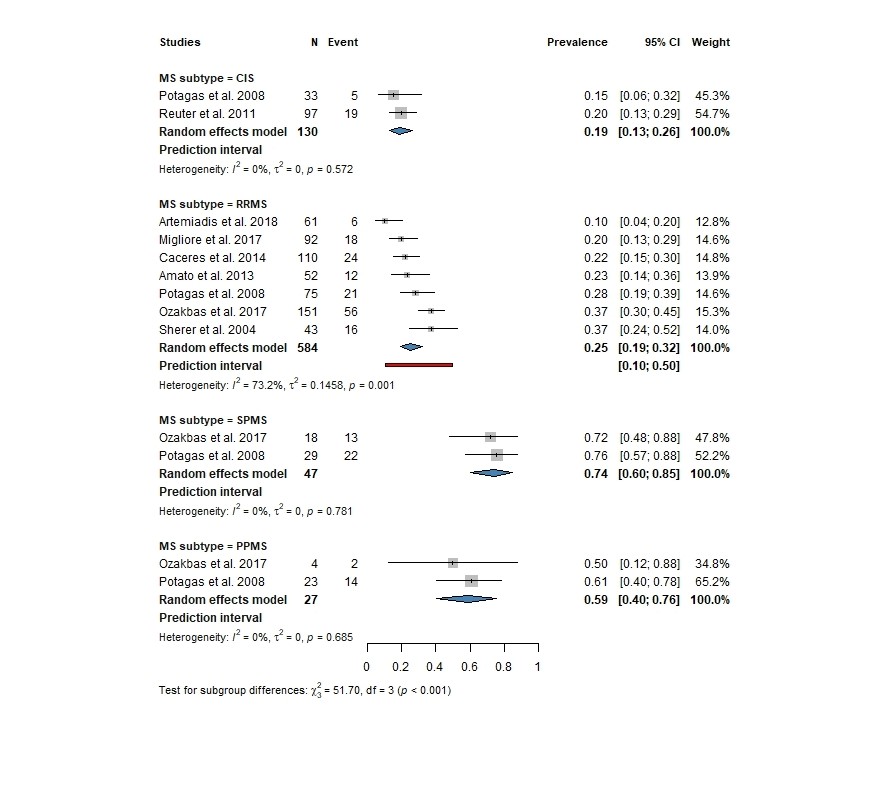
**

**eFigure 3.** Individual plot of information processing speed (IPS)/attention DSI (domain-specific impairment) prevalence rates measured by Symbol Digit Modalities Test (SDMT) at the cut-off of the score below the 5th percentile of the normative values.

**
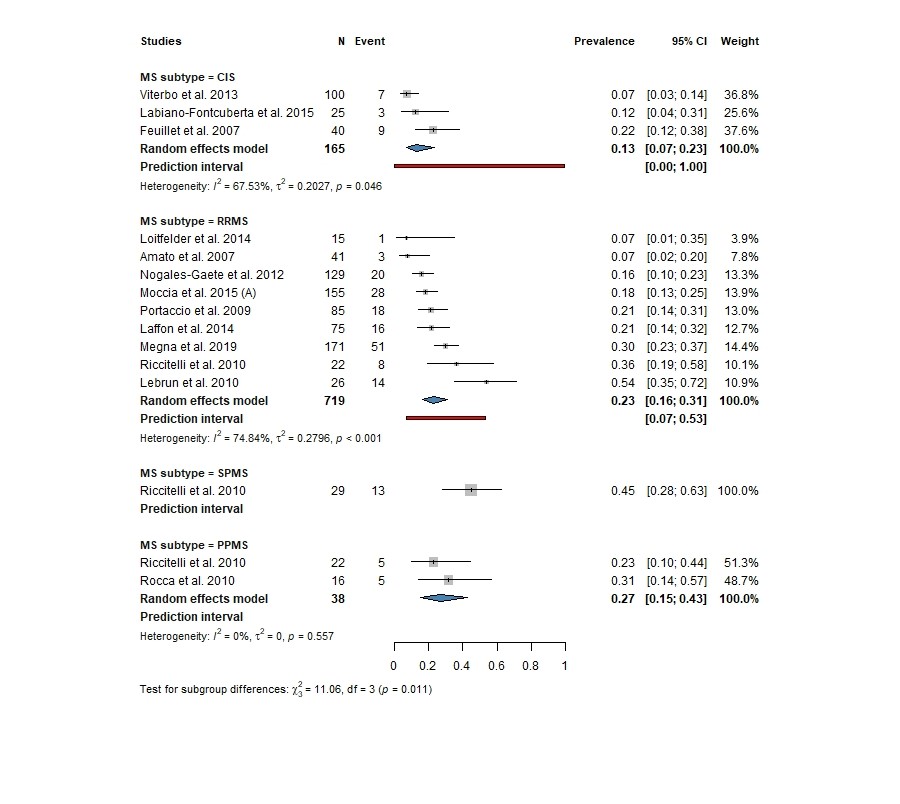
**

**eFigure 4.** Individual plot of working memory DSI (domain-specific impairment) prevalence rates measured by Paced Auditory Serial Addition Test (PASAT 3) at the cut-off of 2.0 SD below the normative value.

**
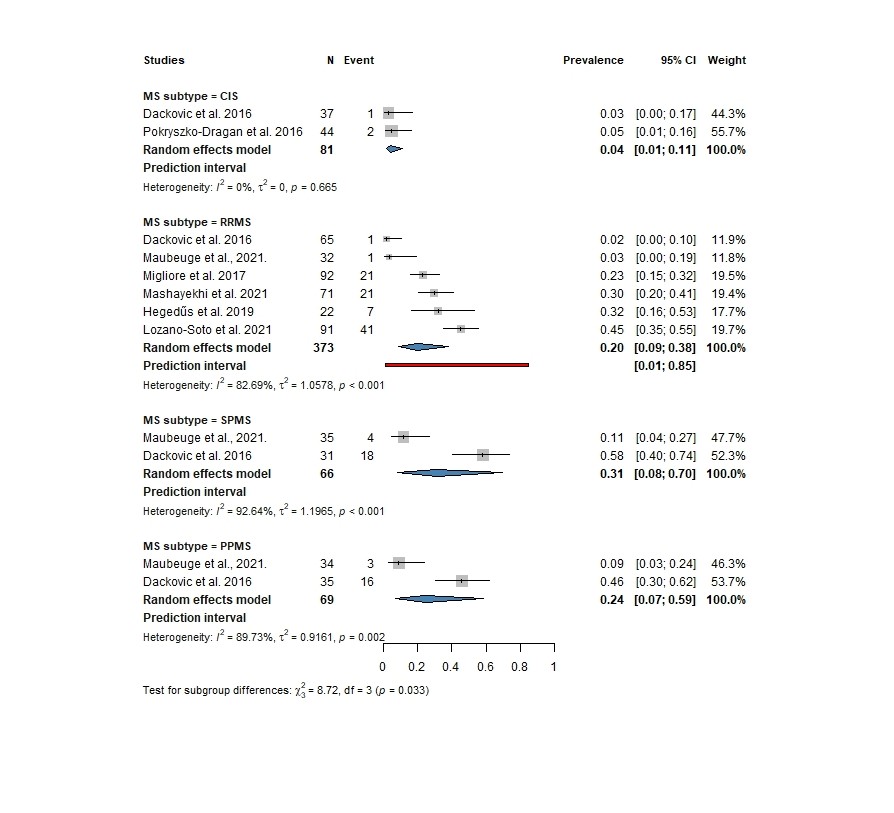
**

**eFigure 5.** Individual plot of working memory DSI (domain-specific impairment) prevalence rates measured by Paced Auditory Serial Addition Test (PASAT 3) at the cut-off of 1.5 SD below the normative value.

**
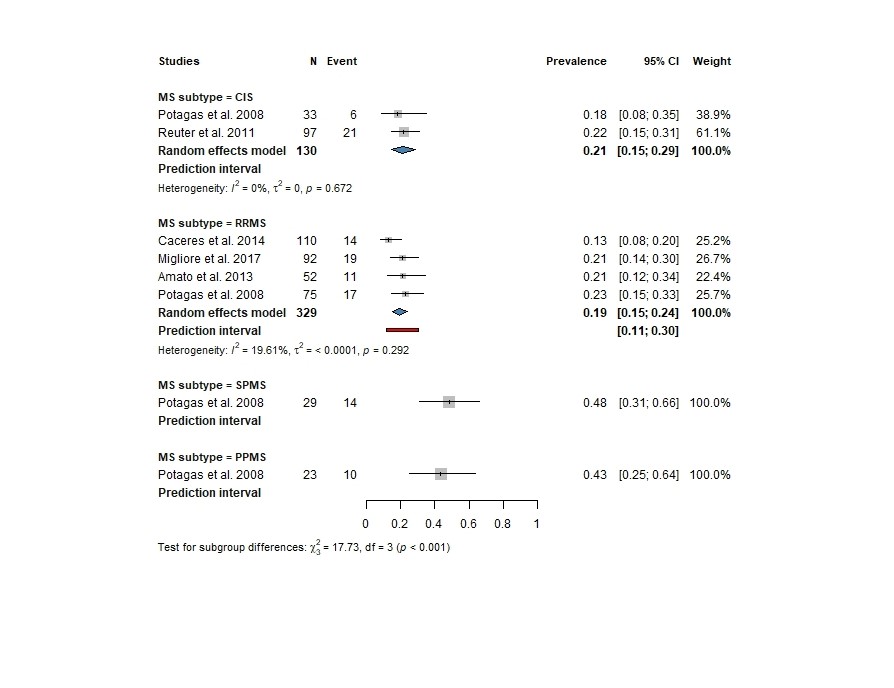
**

**eFigure 6.** Individual plot of working memory DSI (domain-specific impairment) prevalence rates measured by Paced Auditory Serial Addition Test (PASAT 3) at the cut-off of the score below the 5th percentile of the normative values.

**
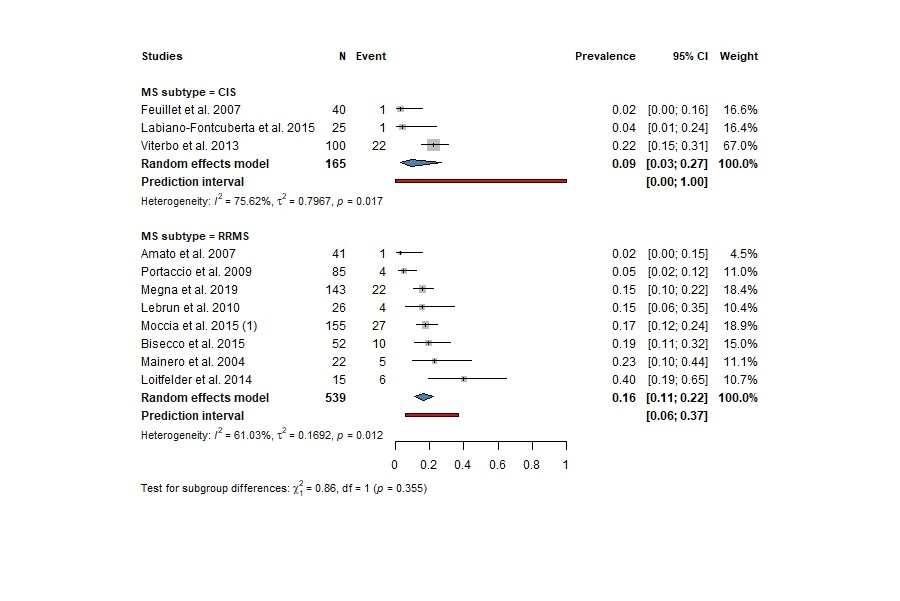
**

**eFigure 7.** Individual plot of language function/verbal fluency DSI (domain-specific impairment) prevalence rates measured by Word List Generation Test (WLG) at the cut-off of 2.0 SD below the normative value.

**
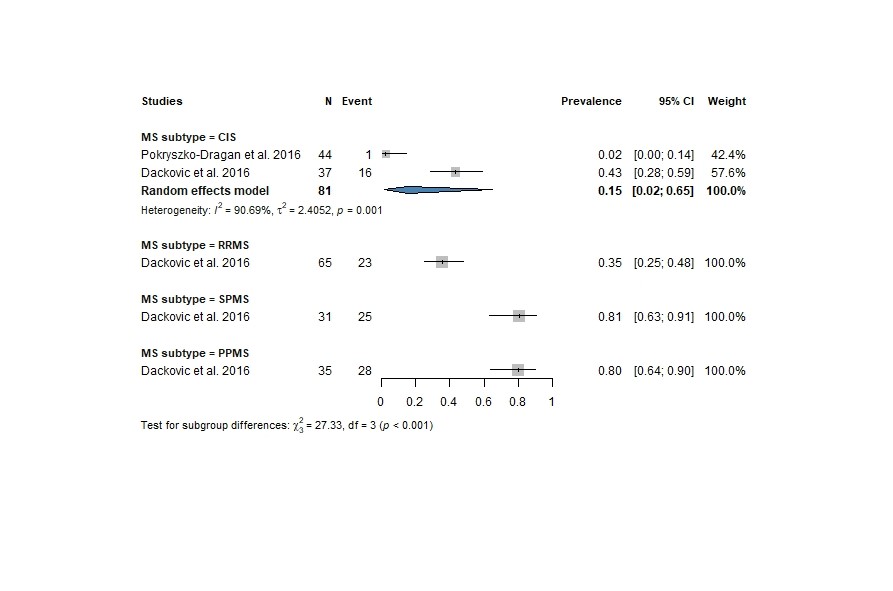
**

**eFigure 8.** Individual plot of language function/verbal fluency DSI (domain-specific impairment) prevalence rates measured by Word List Generation Test (WLG) at the cut-off of 1.5 SD below the normative value.

**
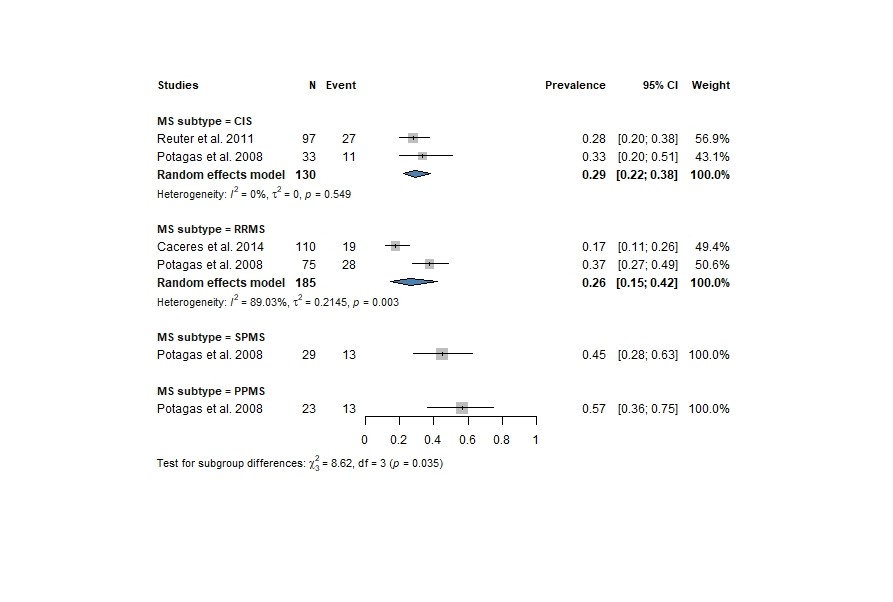
**

**eFigure 9.** Individual plot of language function/verbal fluency DSI (domain-specific impairment) prevalence rates measured by Word List Generation Test (WLG) at the cut-off of the score below the 5th percentile of the normative values.

**
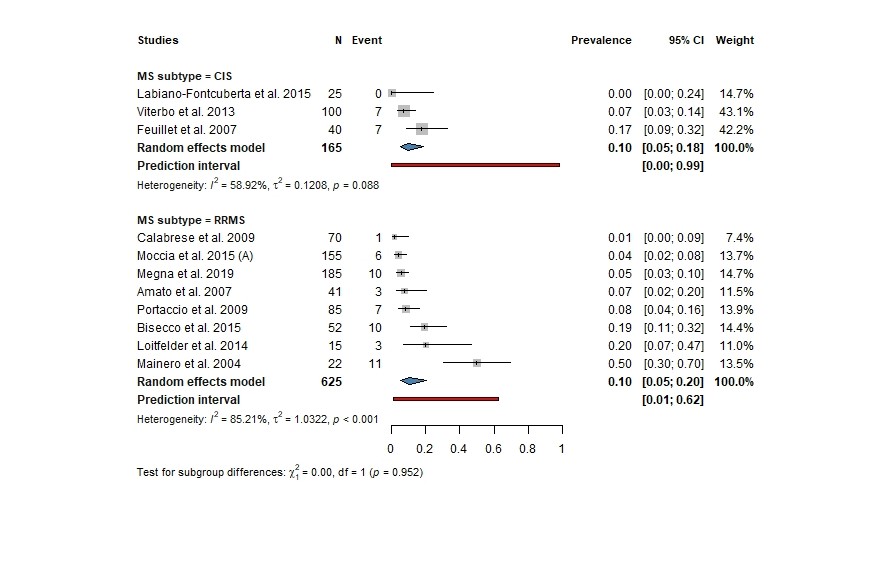
**

**eFigure 10.** Individual plot of perceptual/visuospatial memory DSI (domain-specific impairment) prevalence rates measured by 10/36 Spatial Recall Test (10/36 SPART) at the cut-off of 2.0 SD below the normative value.

**
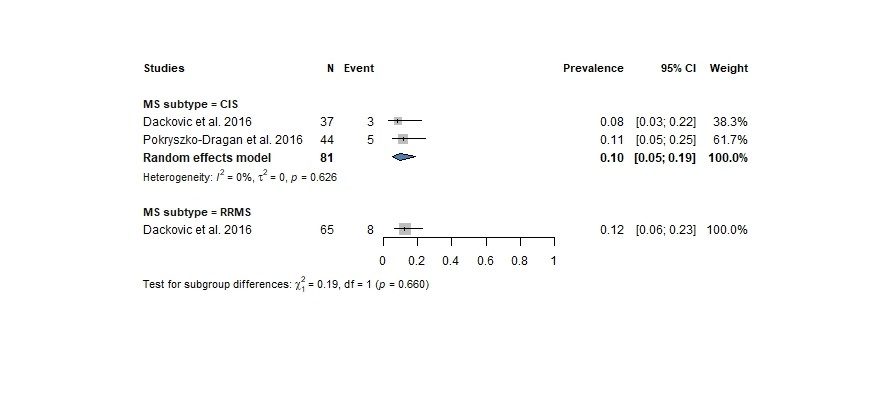
**

**eFigure 11.** Individual plot of perceptual/visuospatial memory DSI (domain-specific impairment) prevalence rates measured by 10/36 Spatial Recall Test (10/36 SPART) at the cut-off of 1.5 SD below the normative value.

**
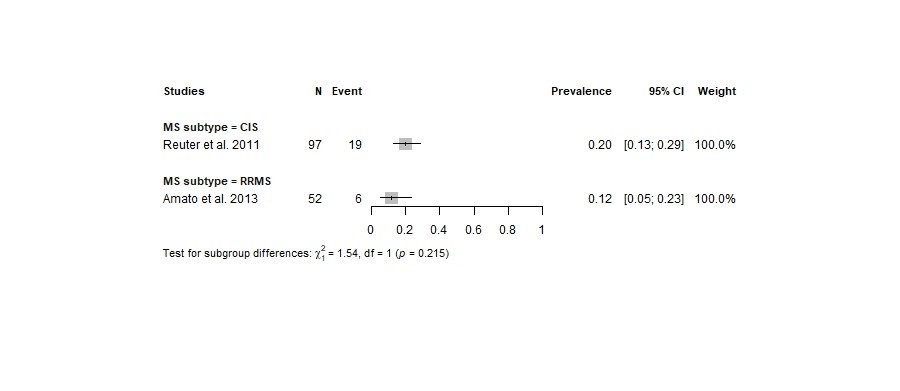
**

**eFigure 12.** Individual plot of perceptual/visuospatial memory DSI (domain-specific impairment) prevalence rates measured by 10/36 Spatial Recall Test (10/36 SPART) at the cut-off of the score below the 5th percentile of the normative values.

**
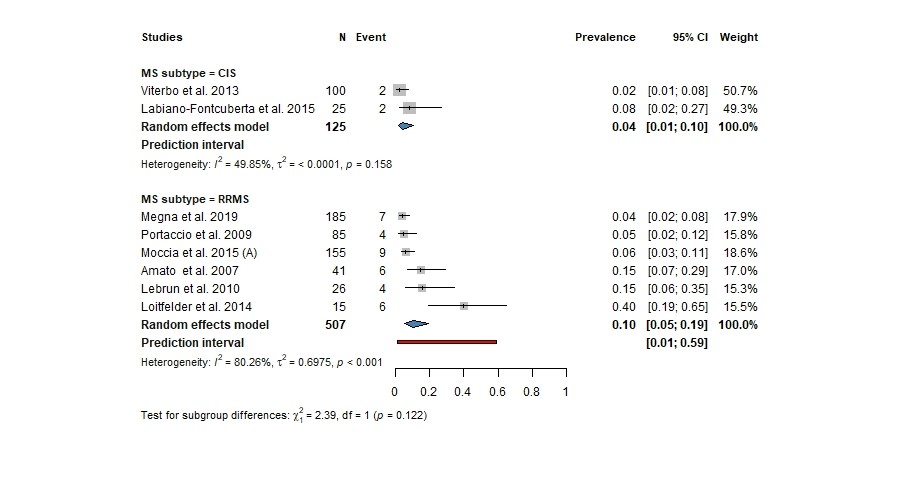
**

**eFigure 13.** Individual plot of perceptual/visuospatial memory delayed recall DSI (domain-specific impairment) prevalence rates measured by 10/36 Spatial Recall Test - DR (10/36 SPART-DR) at the cut-off of 2.0 SD below the normative value.

**
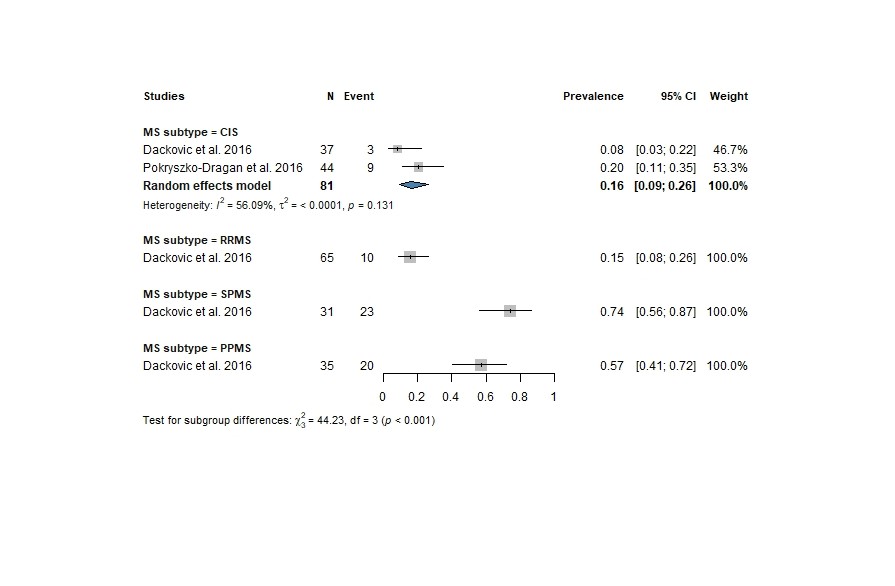
**

**eFigure 14.** Individual plot of perceptual/visuospatial memory delayed recall DSI (domain-specific impairment) prevalence rates measured by 10/36 Spatial Recall Test – DR (10/36 SPART-DR) at the cut-off of 1.5 SD below the normative value.

**
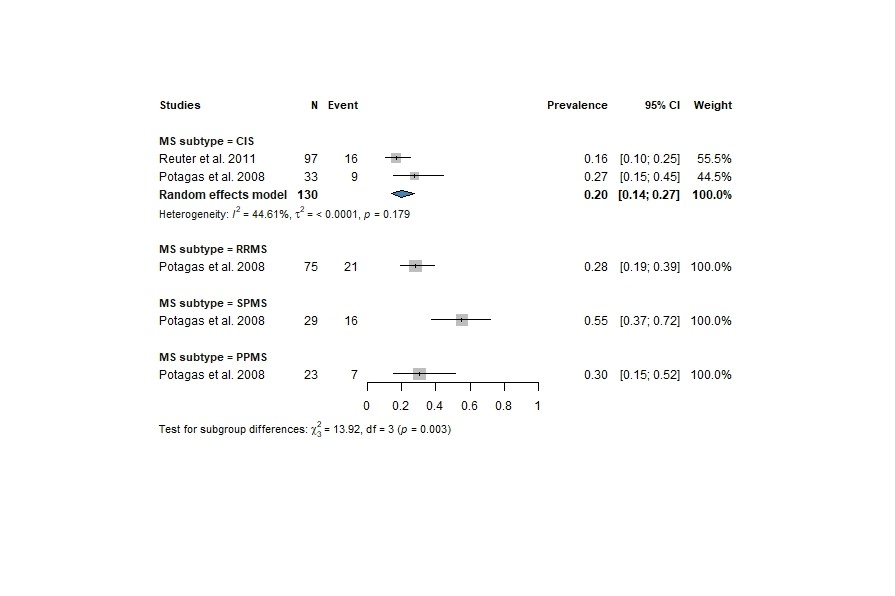
**

**eFigure 15.** Individual plot of perceptual/visuospatial memory delayed recall DSI (domain-specific impairment) prevalence rates measured by 10/36 Spatial Recall Test - DR (10/36 SPART-DR) at the cut-off of the score below the 5th percentile of the normative values.

**
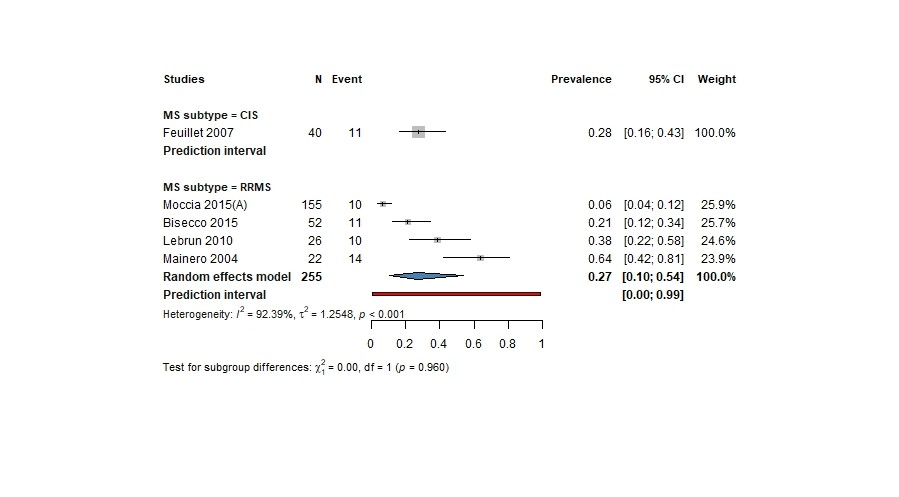
**

**eFigure 16.** Individual plot of learning and verbal memory DSI (domain-specific impairment) prevalence rates measured by Selective Reminding Test (SRT) at the cut-off of 2.0 SD below the normative value.

**
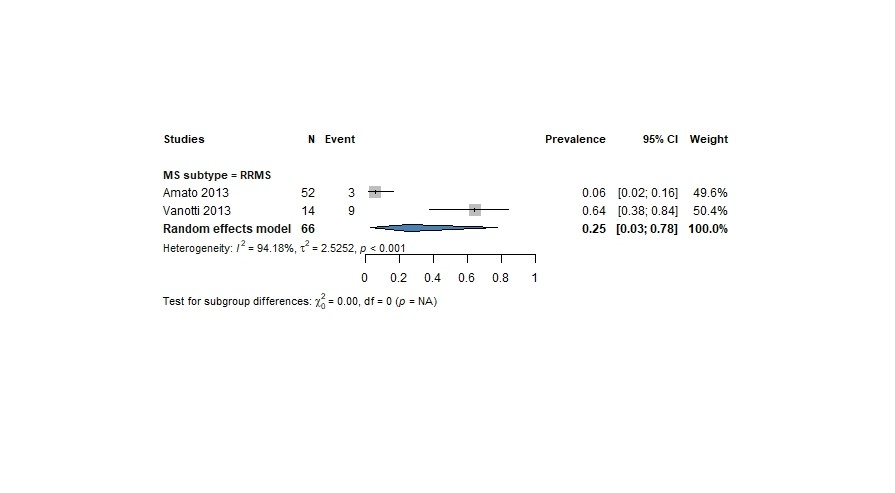
**

**eFigure 17.** Individual plot of learning and verbal memory DSI (domain-specific impairment) prevalence rates measured by Selective Reminding Test (SRT) at the cut-off of the score below the 5th percentile of the normative values.

**
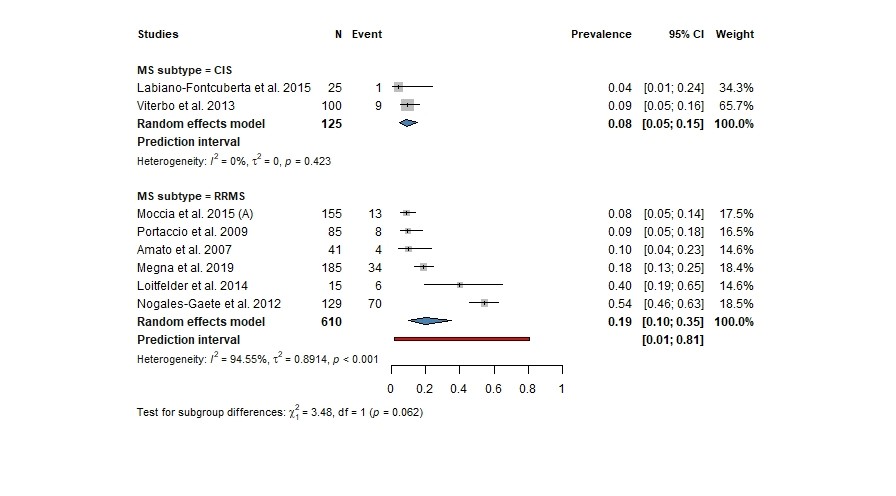
**

**eFigure 18.** Individual plot of learning and verbal memory delayed recall DSI (domain-specific impairment) prevalence rates measured by Selective Reminding Test – DR (SRT-DR) at the cut-off of 2.0 SD below the normative value.

**
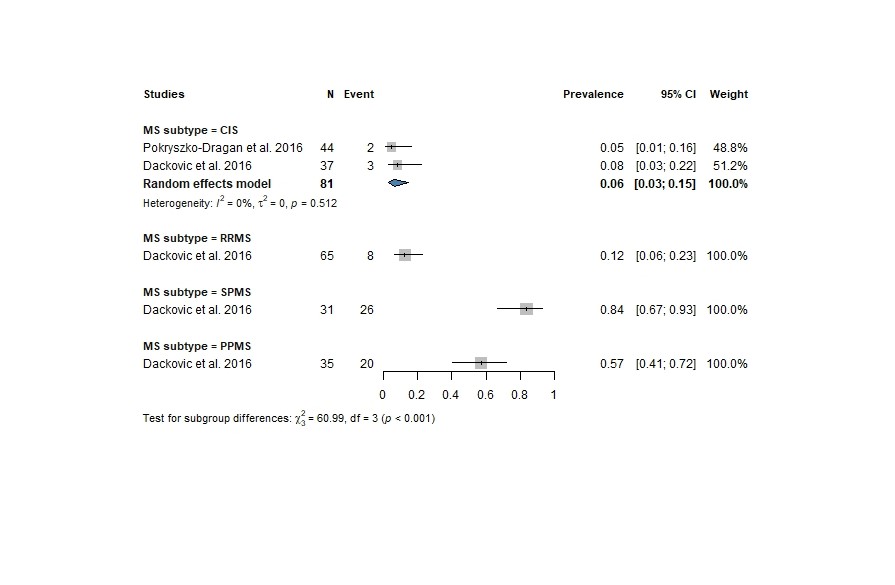
**

**eFigure 19.** Individual plot of learning and verbal memory delayed recall DSI (domain-specific impairment) prevalence rates measured by Selective Reminding Test – DR (SRT-DR) at the cut-off of 1.5 SD below the normative value.

**
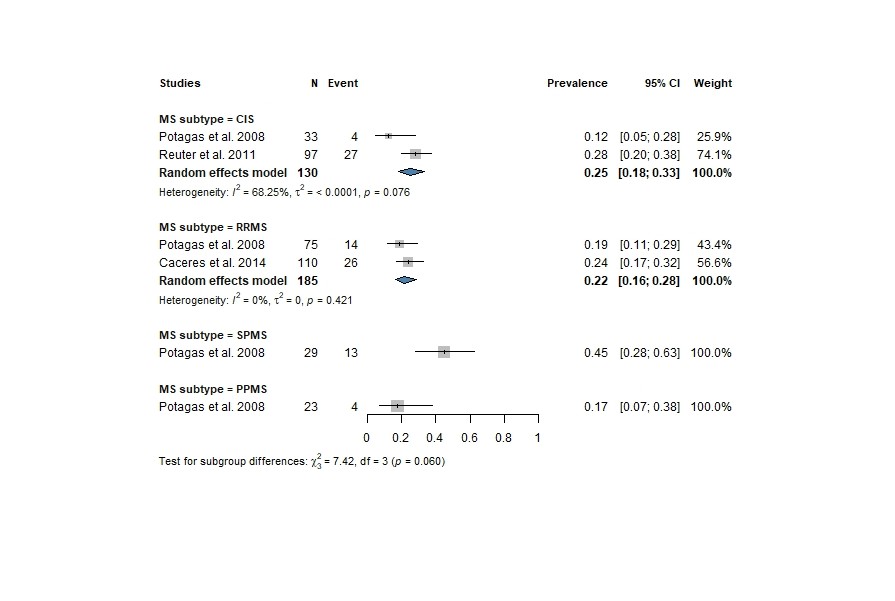
**

**eFigure 20.** Individual plot of learning and verbal memory delayed recall DSI (domain-specific impairment) prevalence rates measured by Selective Reminding Test – DR (SRT-DR) at the cut-off of the score below the 5th percentile of the normative values.

**
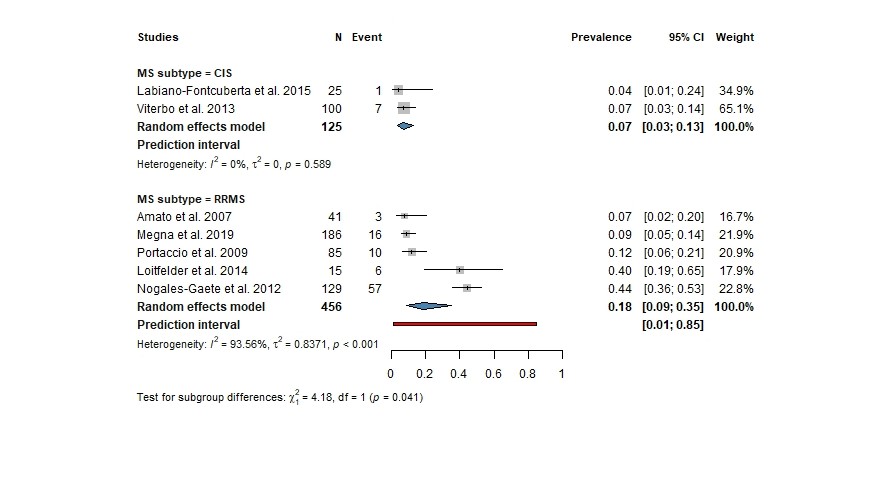
**

**eFigure 21.** Individual plot of learning and verbal memory long-term storage DSI (domain-specific impairment) prevalence rates measured by Selective Reminding Test – LTS (SRT-LTS) at the cut-off of 2.0 SD below the normative value.

**
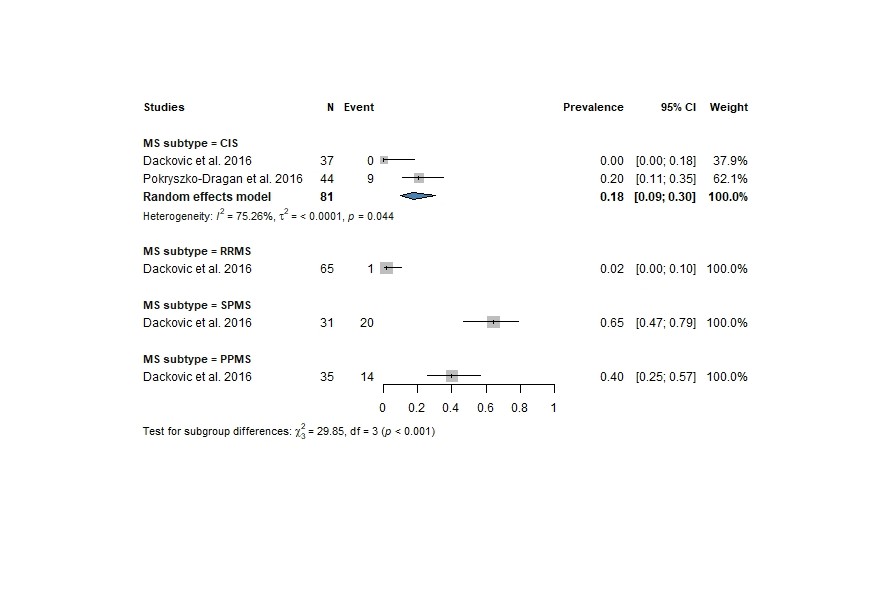
**

**eFigure 22.** Individual plot of learning and verbal memory long-term storage DSI (domain-specific impairment) prevalence rates measured by Selective Reminding Test – LTS (SRT-LTS) at the cut-off of 1.5 SD below the normative value.

**
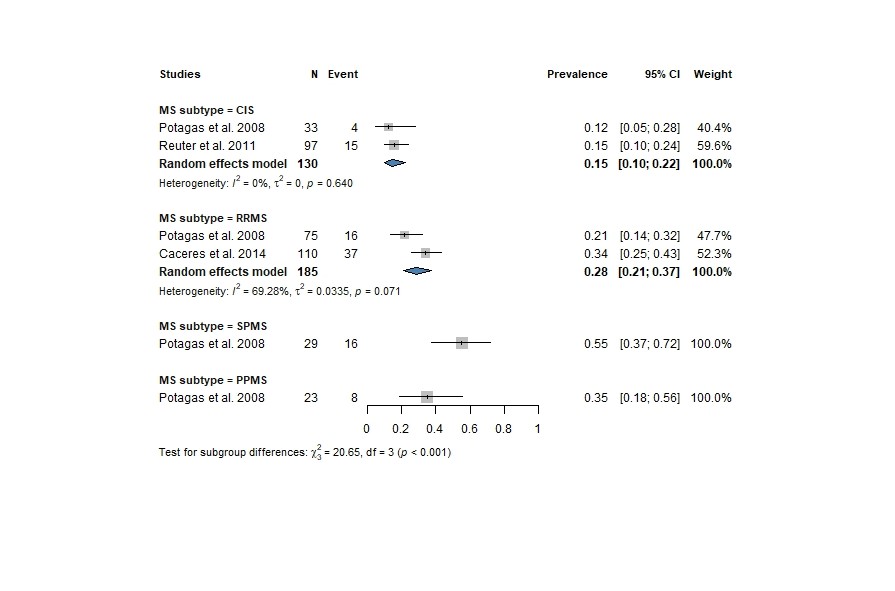
**

**eFigure 23.** Individual plot of learning and verbal memory long-term storage DSI (domain-specific impairment) prevalence rates measured by Selective Reminding Test – LTS (SRT-LTS) at the cut-off of the score below the 5th percentile of the normative values.

**
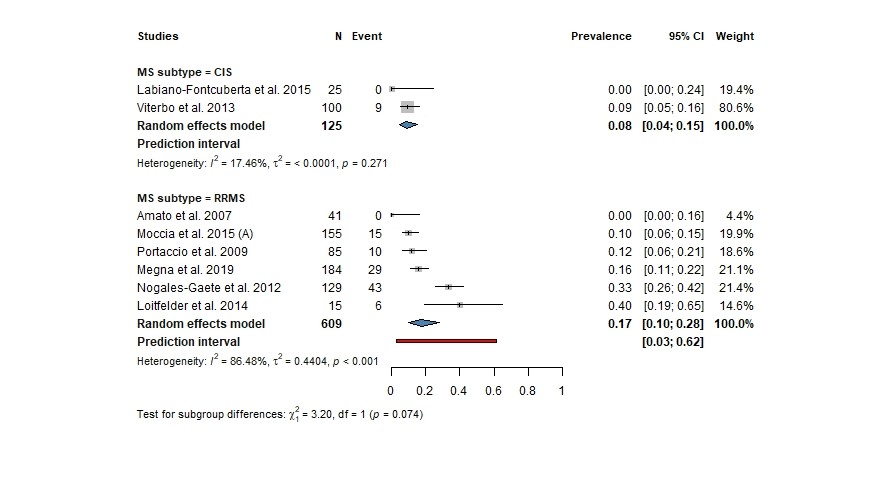
**

**eFigure 24.** Individual plot of learning and verbal memory consistent long-term retrieval DSI (domain-specific impairment) prevalence rates measured by Selective Reminding Test – CLTR (SRT-CLTR) at the cut-off of 2.0 SD below the normative value.

**
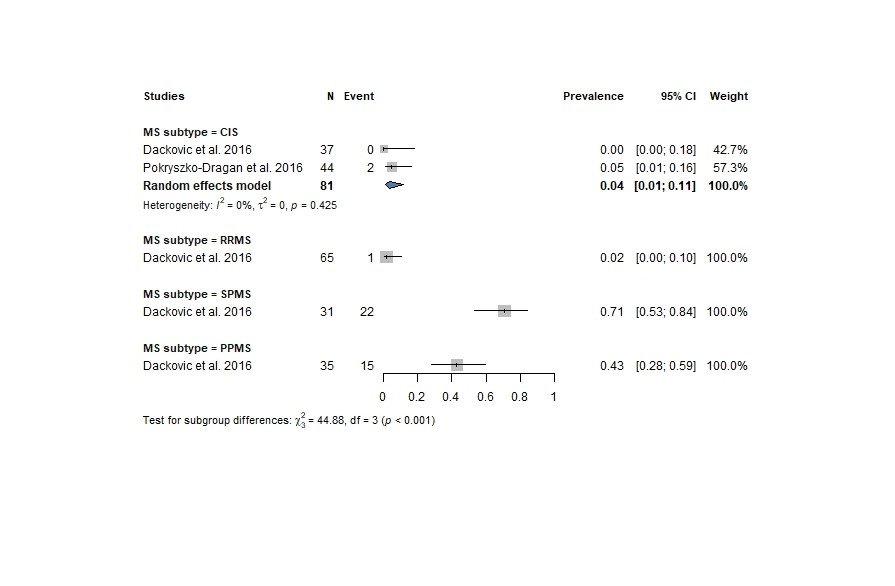
**

**eFigure 25.** Individual plot of learning and verbal memory consistent long-term retrieval DSI (domain-specific impairment) prevalence rates measured by Selective Reminding Test – CLTR (SRT-CLTR) at the cut-off of 1.5 SD below the normative value.

**
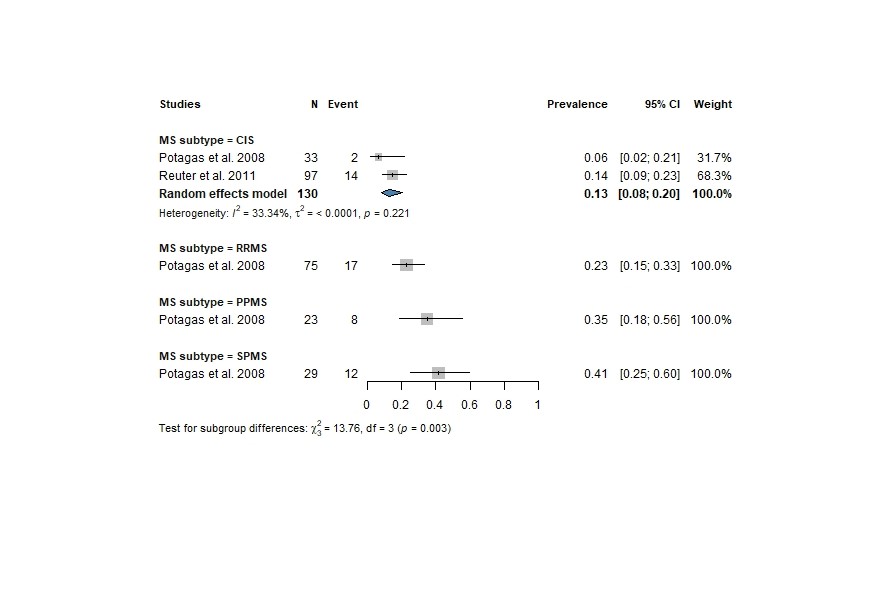
**

**eFigure 26.** Individual plot of learning and verbal memory consistent long-term retrieval DSI (domain-specific impairment) prevalence rates measured by Selective Reminding Test – CLTR (SRT-CLTR) at the cut-off of the score below the 5th percentile of the normative values.

*Note:* Not enough data were available for statistical analysis of Selective Reminding Test (SRT) at the cut-off of 1.5 SD below the normative value.

**Appendix 8., Assessment of risk of bias for each included study (listed according to the criteria of the “JBI Quality Assessment Tool for Prevalence Studies”)**^7^:


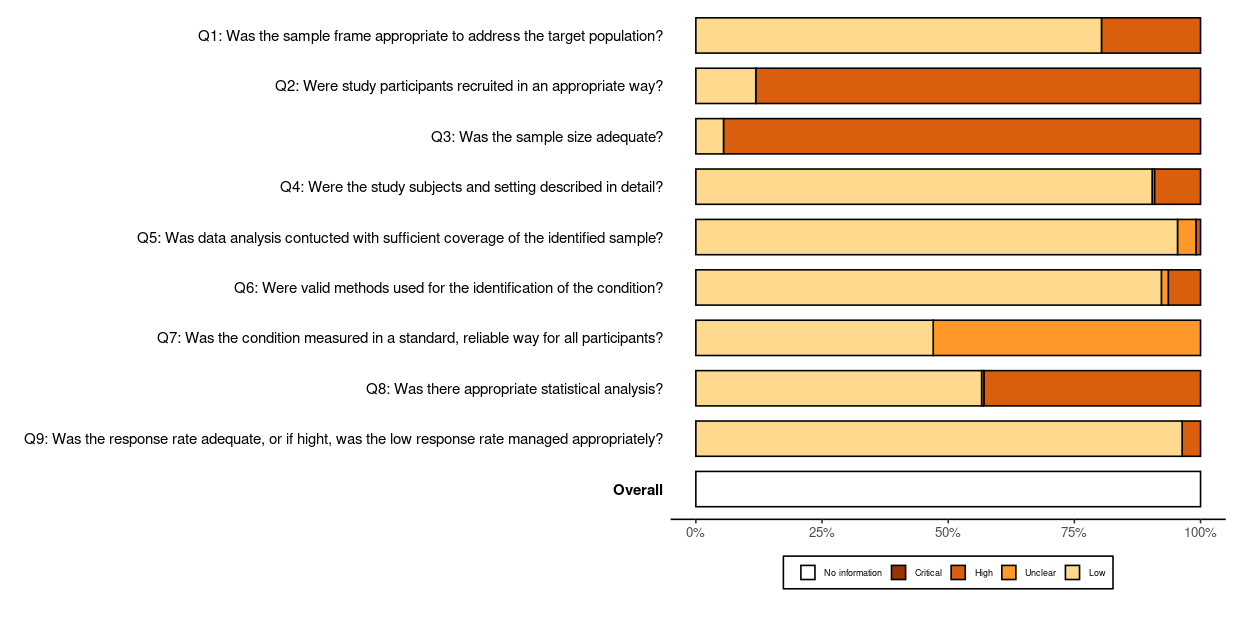


**eFigure 27.** Assessment of risk of bias for each included study (Summary plot).

Quality evaluation based on the "JBI Quality Assessment Tool for Prevalence Studies" criteria, examines the transparency of evidence synthesis results and findings along 9 aspects.

The first three questions refer the possibility of „selection bias” and „performance bias”. As 67% of the primary outcomes were found to be high-risk, there is a possibility of selection bias.

Q4 refers mainly to the level of details about the data reported in the studies (correlated with „reporting bias”): in this respect, our meta-analysis cannot be considered as having a high error rate: only 8.8% of the primary outcomes were found to be at high risk.

Q5,Q6, Q7 questions refer the possibility of „detection bias”. At this point, with a high risk rate of only 2.5%, the resulting margin of error is overall very low, and this is one of the main advantages of our meta-analysis: the uniform, but separated detection criteria that we used.

For Q8, which is primarily concerned with statistical evaluation, a study was considered low-risk if not only the percentage but also the exact number of patients impaired in the cognitive domain was mentioned and the exact definition of the cut-off value was described.

Q9 question is related to „attriton bias”, the probability of error is very low: only 3.7% of the primary outcomes were classified as high risk.


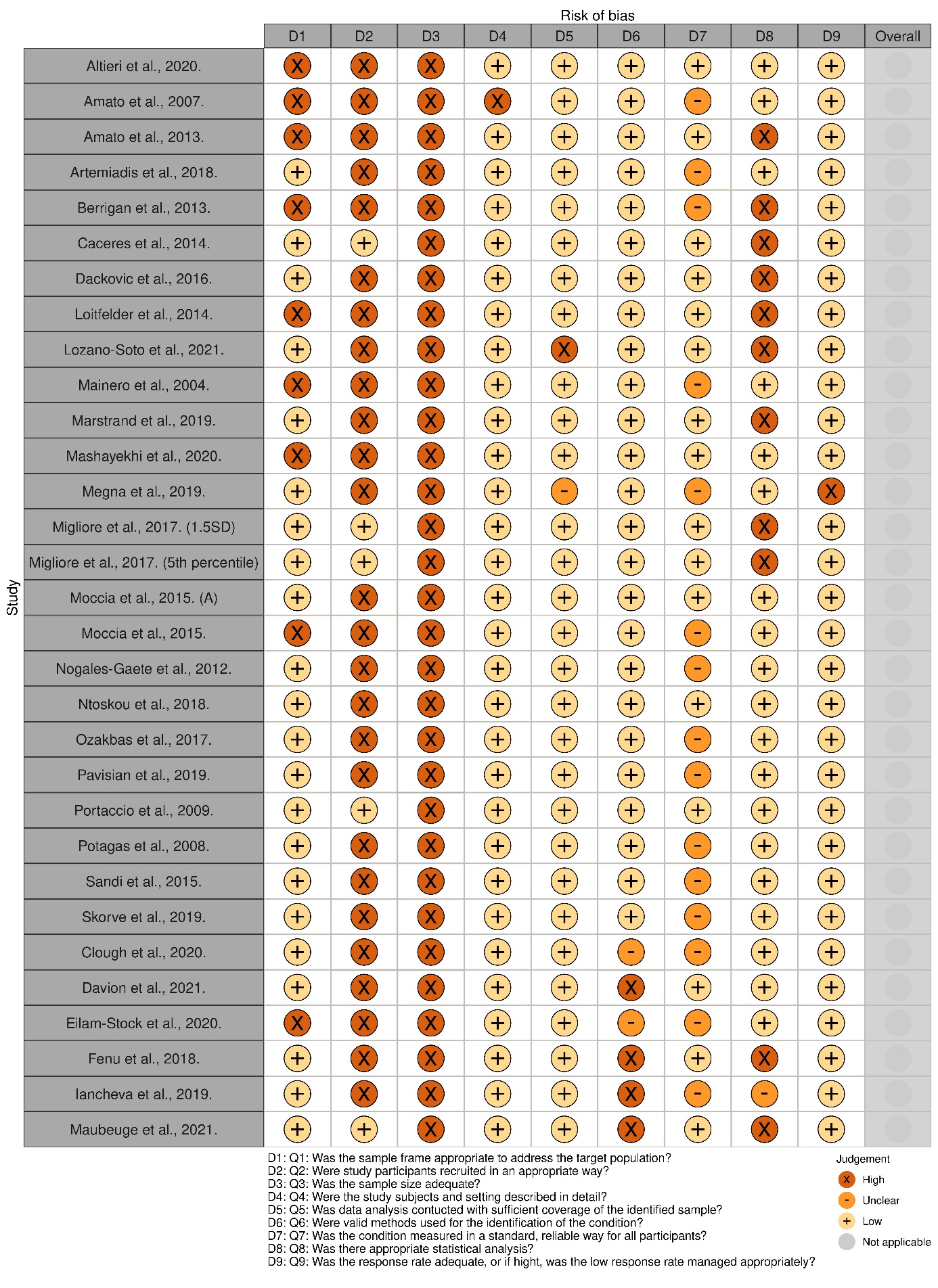


**eFigure 28.** Assessment of risk of bias of each included study with the outcome of information processing speed (IPS)/attention cognitive domain measured by SDMT (Symbol Digit Modalities Test) in relapsing-remitting multiple sclerosis (RRMS) patients.


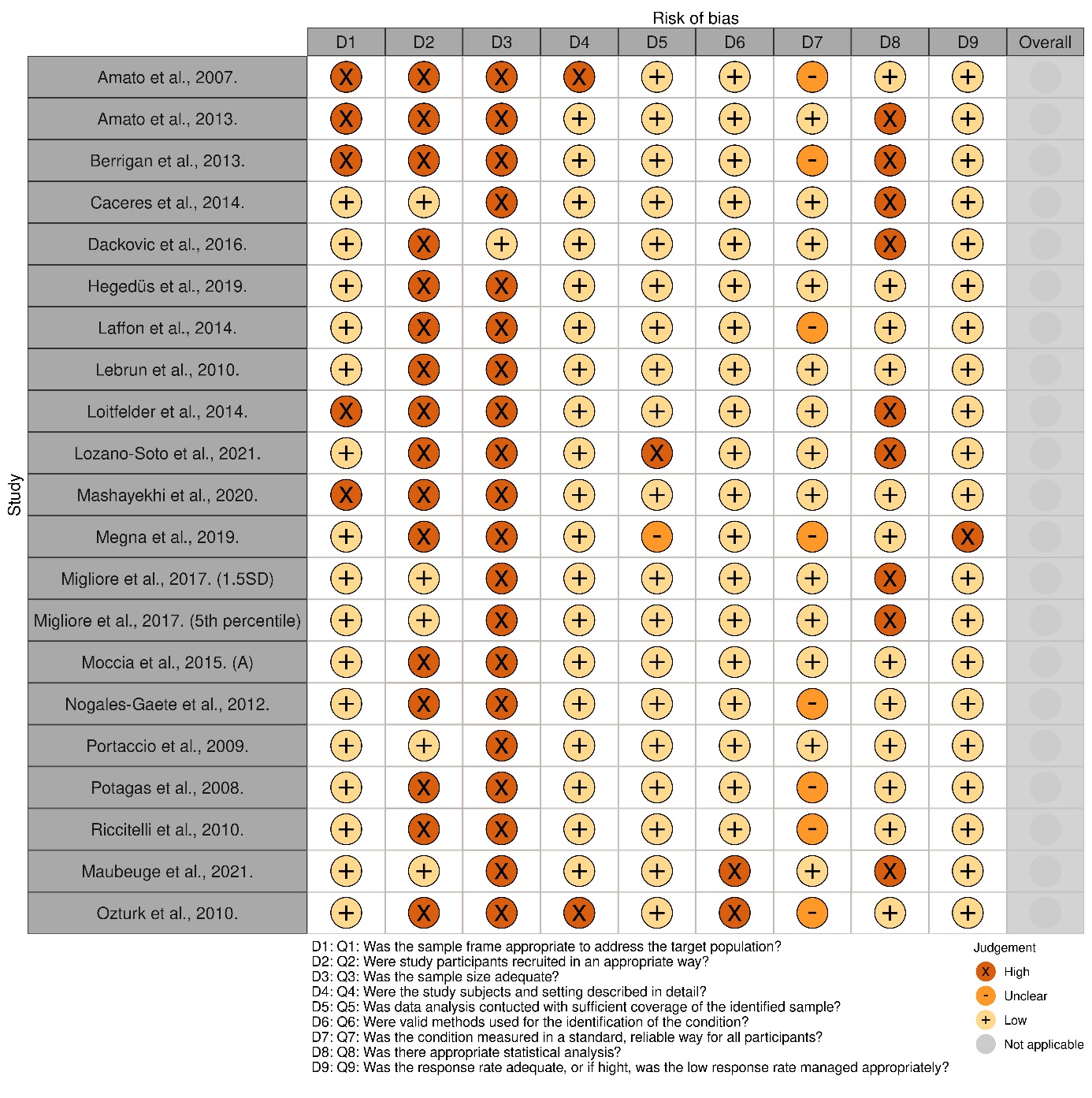


**eFigure 29.** Assessment of risk of bias of each included study with the outcome of working memory cognitive domain measured by PASAT3 (Paced Auditory Serial Addition Test) in relapsing-remitting (RRMS) patients.

**
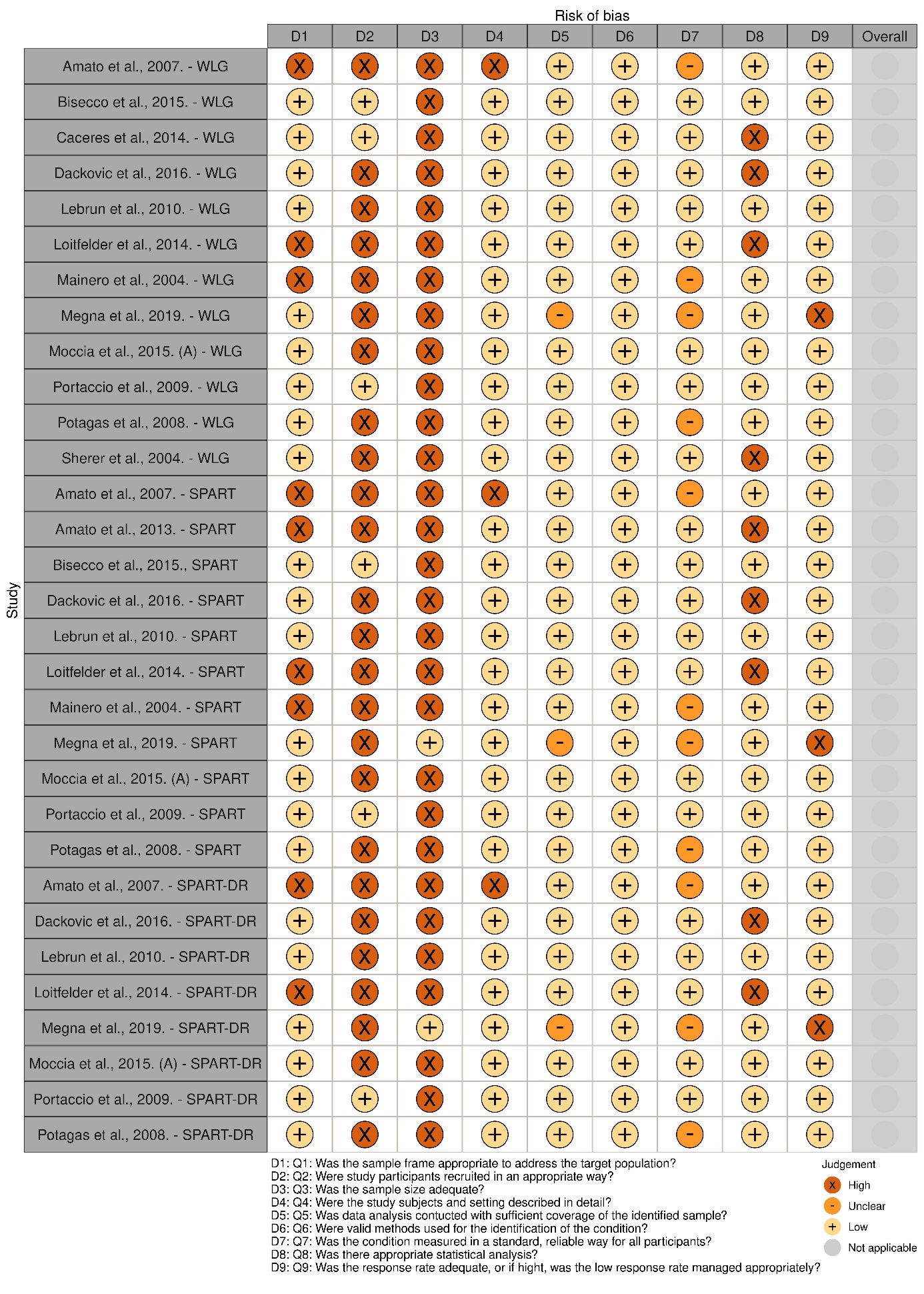
**

**eFigure 30.** Assessment of risk of bias of each included study with the outcome of language function/verbal fluency, perceptual-motor/visuospatial memory and perceptual-motor/visuospatial memory delayed recall cognitive domains measured by WLG (Word List Generation), SPART (10/36 Spatial Recall Test) and SPART-DR (10/36 Spatial Recall Test – Delayed Recall) in relapsing-remitting multiple sclerosis (RRMS) patients.

**
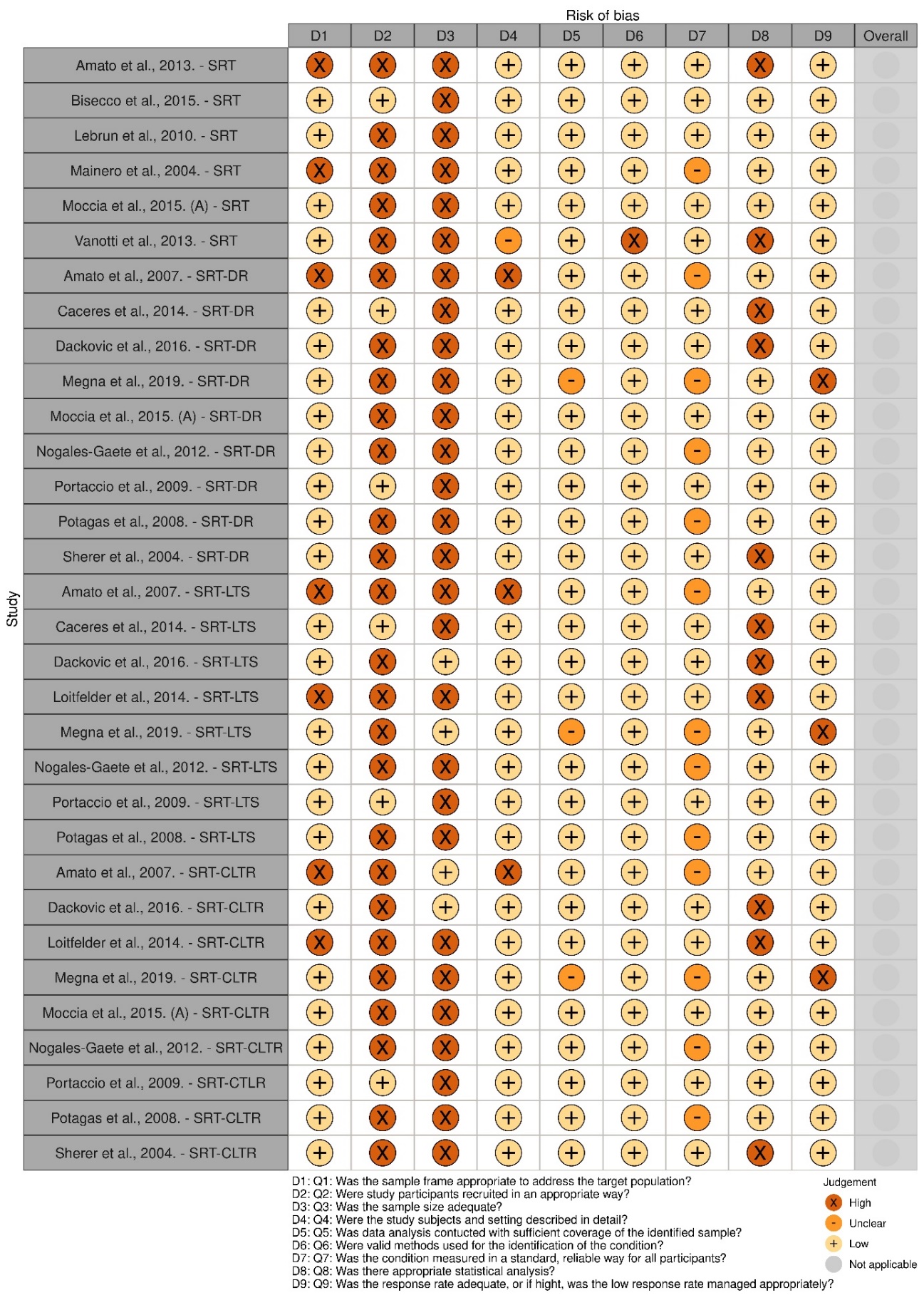
eFigure 31.** Assessment of risk of bias of each included study with the outcome of learning and verbal memory, learning and verbal memory delayed recall, long-term storage and consistent long-term retrieval cognitive domains measured by SRT (Selective Reminding Test), SRT-DR (Selective Reminding Test – Delayed Recall), SRT-LTS (Selective Reminding Test - Long-Term Storage), SRT-CLTR (Selective Reminding Test - Consistent Long-Term Retrieval) in relapsing-remitting (RRMS) patients.

**
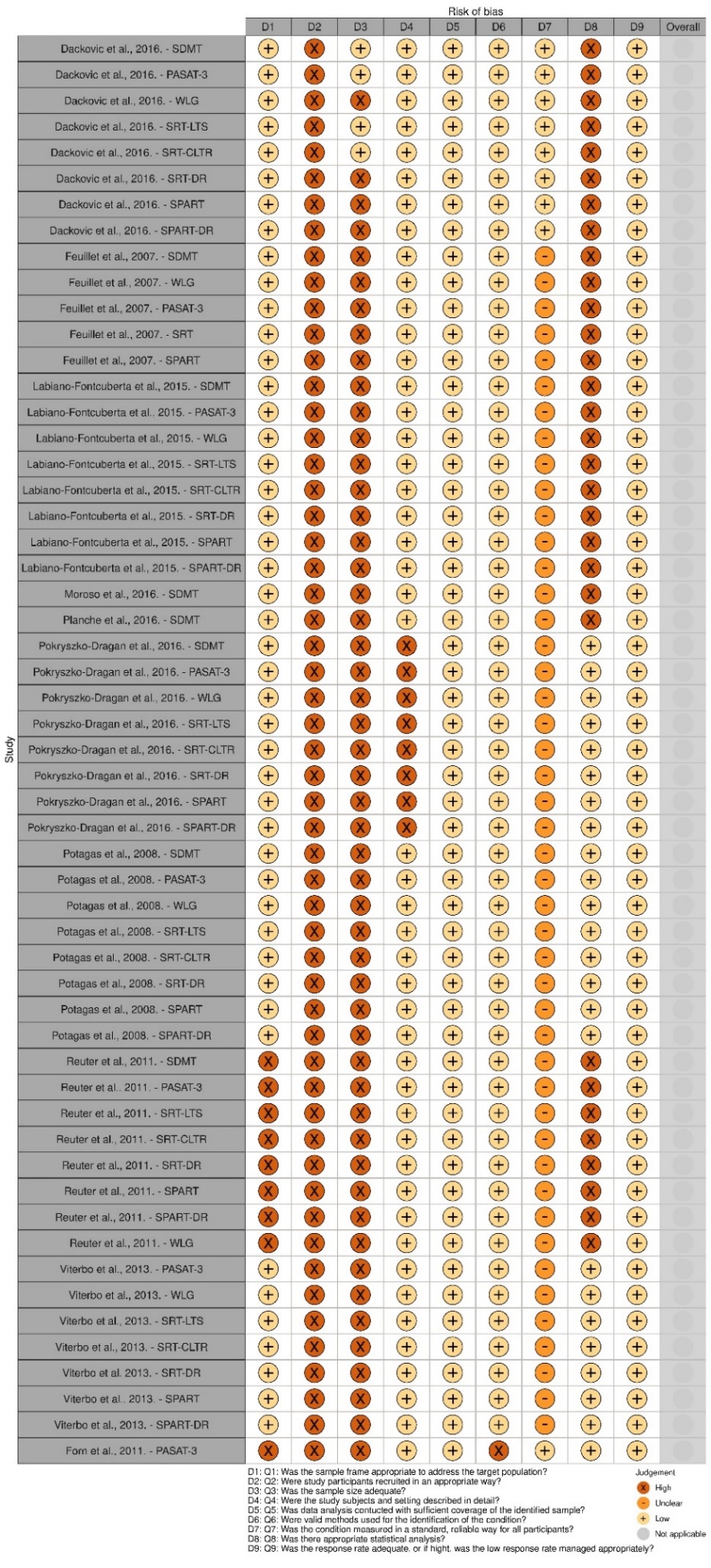
**

**eFigure 32.** Assessment of risk of bias of each included study with the outcome of all investigated cognitive domains in clinically isolated (CIS) patients.

**
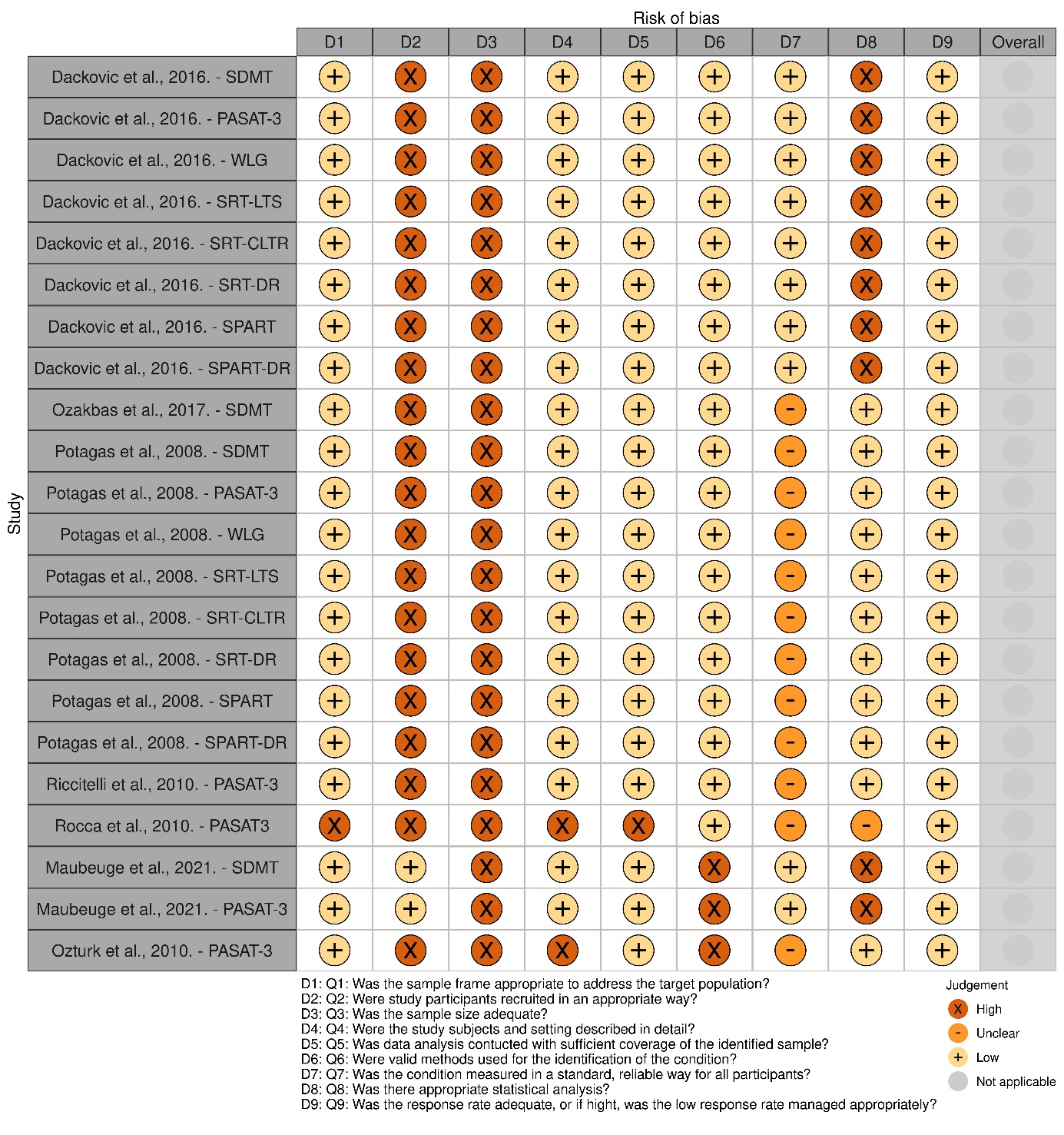
**

**eFigure 33.** Assessment of risk of bias of each included study with the outcome of all investigated cognitive domains in primary progressive multiple sclerosis (PPMS) patients.

**
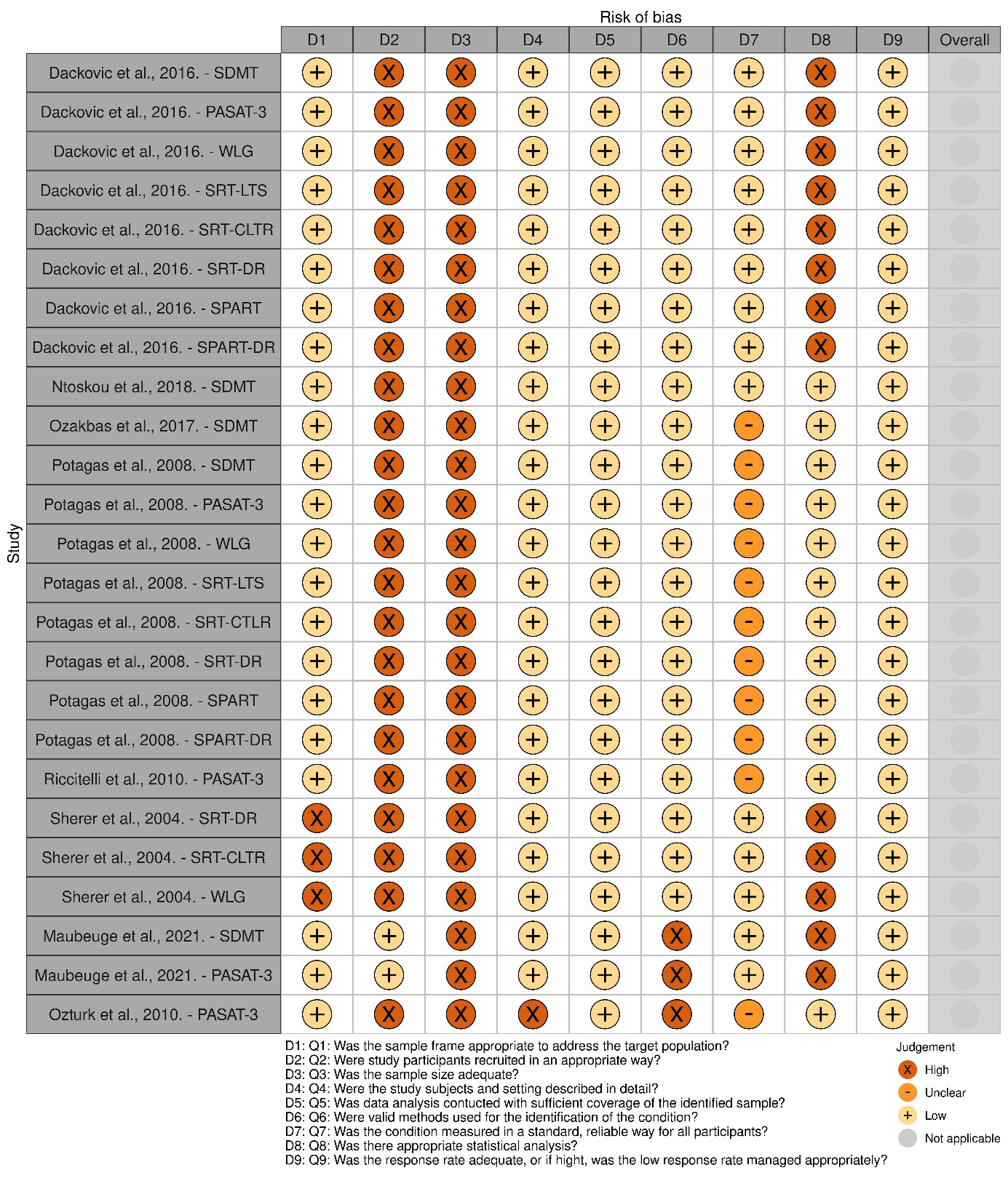
**

**eFigure 34.** Assessment of risk of bias of each included study with the outcome of all investigated cognitive domains in secondary progressive multiple sclerosis (SPMS) patients.

**Appendix 9.,** **Ratings of the quality of the evidence:**

**
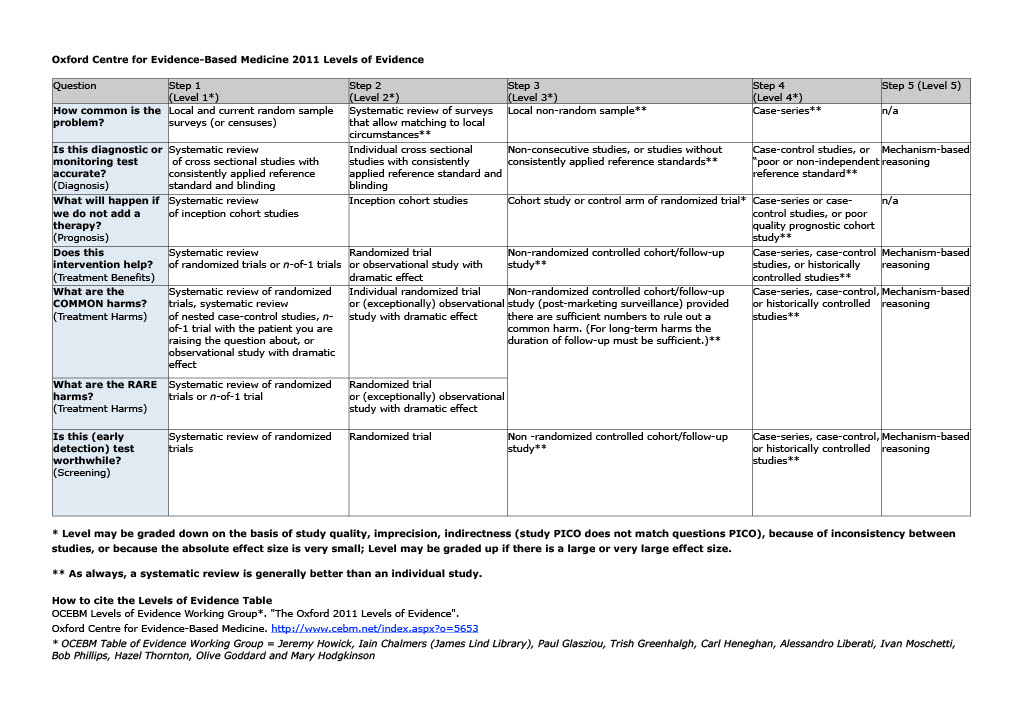
**

**eFigure 35.** Modified Oxford Centre for Evidence-Based Medicine Levels of Evidence (2011.)^8,9^

Classification acording to the table: Level 2.

**References:**

1. Page MJ, McKenzie JE, Bossuyt PM, Boutron I, Hoffmann TC, Mulrow CD, et al. The PRISMA 2020 statement: an updated guideline for reporting systematic reviews. BMJ 2021;372:n71. doi: 10.1136/bmj.n71 For more information, visit: <http://www.prisma-statement.org/>
2. McDonald WI, Compston A, Edan G,et al. Recommended diagnostic criteria for multiple sclerosis: guidelines from the International Panel on the diagnosis of multiple sclerosis. Ann Neurol. 2001 Jul;50(1):121-7. doi: 10.1002/ana.1032. PMID: 11456302.
3. Polman CH, Reingold SC, Edan G, et al. Diagnostic criteria for multiple sclerosis: 2005 revisions to the "McDonald Criteria". Ann Neurol. 2005 Dec;58(6):840-6. doi: 10.1002/ana.20703. PMID: 16283615.
4. Polman CH, Reingold SC, Banwell B,et al. Diagnostic criteria for multiple sclerosis: 2010 revisions to the McDonald criteria. Ann Neurol. 2011 Feb;69(2):292-302. doi: 10.1002/ana.22366. PMID: 21387374; PMCID: PMC3084507.
5. Thompson AJ, Banwell BL, Barkhof F, et al. Diagnosis of multiple sclerosis: 2017 revisions of the McDonald criteria. Lancet Neurol. 2018 Feb;17(2):162-173. doi: 10.1016/S1474-4422(17)30470-2. Epub 2017 Dec 21. PMID: 29275977.
6. Migliore S, Ghazaryan A, Simonelli I, et al. Cognitive Impairment in Relapsing-Remitting Multiple Sclerosis Patients with Very Mild Clinical Disability. Behav Neurol. 2017;2017:7404289. doi: 10.1155/2017/7404289. Epub 2017 Aug 15. PMID: 28912625; PMCID: PMC5574272.
7. McGuinness, LA, Higgins, JPT. Risk-of-bias VISualization (robvis): An R package and Shiny web app for visualizing risk-of-bias assessments. Res Syn Meth. 2020; 1- 7. <https://doi.org/10.1002/jrsm.1411>
8. Jeremy Howick, Iain Chalmers, Paul Glasziou, Trish Greenhalgh, Carl Heneghan, Alessandro Liberati, Ivan Moschetti, Bob Phillips, and Hazel Thornton. “Explanation of the 2011 Oxford Centre for Evidence-Based Medicine (OCEBM) Levels of Evidence (Background Document)”. Oxford Centre for Evidence-Based Medicine. <https://www.cebm.ox.ac.uk/resources/levels-of-evidence/explanation-of-the-2011-ocebm-levels-of-evidence/>
9. OCEBM Levels of Evidence Working Group*. "The Oxford 2011 Levels of Evidence". Oxford Centre for Evidence-Based Medicine. http://www.cebm.net/index.aspx?o=5653 * OCEBM Table of Evidence Working Group = Jeremy Howick, Iain Chalmers (James Lind Library), Paul Glasziou, Trish Greenhalgh, Carl Heneghan, Alessandro Liberati, Ivan Moschetti, Bob Phillips, Hazel Thornton, Olive Goddard and Mary Hodgkinson
