# Supplementary material for: Domain‐specific cognitive impairment in multiple sclerosis: A systematic review and meta‐analysis
Source: Ann Clin Transl Neurol. 2024 Jan 11;11(3):564–76. doi: 10.1002/acn3.51976 (PMC10963281; doi:10.1002/acn3.51976)
Supplement: Supplementary file 2 — Text S1. [file ACN3-11-564-s001.docx]

Supplemental Files (Supplementary Material):

PRISMA 2020 Checklist and PRISMA 2020 Abstract Checklist (two tables); Search keys; Clinical question and CoCoPop framework for literature searching; Details of the selection and exclusion criteria during the selection process; List of the references for all included studies; Baseline characteristics of the included studies (eTable [1](#F5).); Individual plots of the domain-specific impairment (DSI) in each MS subtype (eFig. [1–26](#F5).); Assessment of risk of bias for each included study (listed per accordance with “JBI Quality Assessment Tool for Prevalence Studies” criteria, eFig. [27–34](#F5).); Ratings of the quality of the evidence (eFig. [35](#F5).)
